# From model to crop: functional characterization of *SPL8* in *M. truncatula* led to genetic improvement of biomass yield and abiotic stress tolerance in alfalfa

**DOI:** 10.1111/pbi.12841

**Published:** 2017-10-17

**Authors:** Jiqing Gou, Smriti Debnath, Liang Sun, Amy Flanagan, Yuhong Tang, Qingzhen Jiang, Jiangqi Wen, Zeng‐Yu Wang

**Affiliations:** ^1^ Noble Research Institute Ardmore OK USA; ^2^ BioEnergy Science Center (BESC) Oak Ridge National Laboratory Oak Ridge TN USA

**Keywords:** *Medicago truncatula*, alfalfa, *Medicago sativa*, forage legume, branching, biomass yield, salt tolerance, drought tolerance

## Abstract

Biomass yield, salt tolerance and drought tolerance are important targets for alfalfa (*Medicago sativa* L.) improvement. *Medicago truncatula* has been developed into a model plant for alfalfa and other legumes. By screening a *Tnt1* retrotransposon‐tagged *M. truncatula* mutant population, we identified three mutants with enhanced branching. Branch development determines shoot architecture which affects important plant functions such as light acquisition, resource use and ultimately impacts biomass production. Molecular analyses revealed that the mutations were caused by *Tnt1* insertions in the *SQUAMOSA PROMOTER BINDING PROTEIN‐LIKE 8* (*SPL8*) gene. The *M*. *truncatula spl8* mutants had increased biomass yield, while overexpression of *SPL8* in *M. truncatula* suppressed branching and reduced biomass yield. Scanning electron microscopy (SEM) analysis showed that *SPL8* inhibited branching by directly suppressing axillary bud formation. Based on the *M. truncatula SPL8* sequence, alfalfa *SPL8* (*MsSPL8*) was cloned and transgenic alfalfa plants were produced. *MsSPL8* down‐regulated or up‐regulated alfalfa plants exhibited similar phenotypes to the *M. truncatula* mutants or overexpression lines, respectively. Specifically, the *MsSPL8* down‐regulated alfalfa plants showed up to 43% increase in biomass yield in the first harvest. The impact was even more prominent in the second harvest, with up to 86% increase in biomass production compared to the control. Furthermore, down‐regulation of *MsSPL8* led to enhanced salt and drought tolerance in transgenic alfalfa. Results from this research offer a valuable approach to simultaneously improve biomass production and abiotic stress tolerance in legumes.

## Introduction

Alfalfa (*Medicago sativa* L.), known as the ‘Queen of Forages’ due to its remarkable adaptability, high biomass yield, exceptional nutritive value and notable capacity for biological nitrogen fixation, is one of the most important and widely cultivated forage crops around the world (Annicchiarico *et al*., 2015; Aung *et al*., 2015; Biazzi *et al*., 2017; Li *et al*., 2008; Russelle *et al*., 2007; Samac *et al*., 2006). In the last century, extensive efforts have been made to improve alfalfa biomass yield, but the achievements have been limited (Aung *et al*., 2015). The biomass yields of first/second harvests of recently released cultivars have not shown improvements compared to previous cultivars released 50 years ago (Volenec *et al*., 2002). Although biomass yield is a complex trait, use of biotechnology for alfalfa yield improvement has achieved success in recent years. For example, overexpression of microR156 in alfalfa increased biomass yield more than 10% (Aung *et al*., 2015). The efficiency of genetic engineering approaches for improving alfalfa biomass yield depends upon the identification of specific genes that control important agronomic traits (Aung *et al*., 2015; Volenec *et al*., 2002). Alfalfa is an obligate outcrossing and tetraploid species. Seeds from a plant are genetically different and heterogeneous, and genome sequence information is limited. Because of its genetic complexity, the identification of agronomic target genes in alfalfa is difficult (Aung *et al*., 2015).

A corresponding model system with simpler genetics has been developed to meet the challenges of modifying this complex agronomic crop. *Medicago truncatula* belongs to the *Trifolieae* tribe that includes major forage legumes such as alfalfa and clovers (*Trifolium* sp.). Plus it has an autogamous mode of reproduction and a short growth cycle, with the added benefit of a small, deeply sequenced, well‐annotated genome (Young *et al*., 2005, 2011). Because of these advantages, *M. truncatula* has been found to be an excellent model for legumes, especially for alfalfa. Various genetic and genomic resources have been developed in *M. truncatula*, including *Tnt1* retrotransposon‐tagged mutants (>22 000 lines and 520 000 random insertions) and fast‐neutron mutants (>117 000 lines) (https://medicago-mutant.noble.org/mutant/index.php), ecotype collections (Cook, 1999), EST and genespace sequencing information (Young and Udvardi, 2009), and the Gene Expression Atlas (https://mtgea.noble.org/v2/index.php). All of these resources dramatically accelerate studies in *M. truncatula* and alfalfa. However, most previous research related primarily to bacterial and mycorrhizal symbioses, leaf development, disease resistance and seed development (Chai *et al*., 2016; Espinoza Ldel *et al*., 2012; Kang *et al*., 2016; Zhou *et al*., 2014). In contrast, studies on the regulation of aerial morphogenesis, especially of shoot architecture development, are very limited (Espinoza Ldel *et al*., 2012; Julier *et al*., 2007).

Branch development (branching) is a key determinant of shoot architecture which affects important plant functions like light acquisition, resource use and ultimately impacts biomass yield. The primary branch arising from the main shoot produces secondary, then tertiary and even higher order branches. Axillary buds are the sole originators of vegetative and floral branches (Domagalska and Leyser, 2011). Axillary buds arise in the leaf axil (the upper side of the region where the leaf joins to the stem) and exhibit two stages, initiation and outgrowth, ultimately forming the various branches (Bennett and Leyser, 2006). In the last three decades, outgrowth of axillary buds has been well characterized due to the identification of many mutations related to bud outgrowth (Costes *et al*., 2014; Domagalska and Leyser, 2011; Gomez‐Roldan *et al*., 2008). These intensive studies regarding the outgrowth of buds have revealed a global and complex regulation network of genetic, hormonal and environmental factors (Domagalska and Leyser, 2011; Guo *et al*., 2013; Kebrom *et al*., 2013; McSteen, 2009; Wang and Li, 2011). In contrast, the initiation of axillary buds appears to be exclusively genetically regulated without any implication of other contributing factors (Kebrom *et al*., 2013). Currently, only a few genes have been identified, including *Lateral suppressor* (*Ls*) and its orthologs [*LATERAL SUPPRESSOR* (*LAS*) and *MONOCULM1* (*MOC1*)] (Cheng *et al*., 2006; Gallavotti *et al*., 2008; Li *et al*., 2003; Schumacher *et al*., 1999) and *LAX PANICLE1* (*LAX1*) and its orthologs [BARREN STALK1 (BA1)] (Gallavotti *et al*., 2004; Komatsu *et al*., 2003; Yang *et al*., 2012).

SQUAMOSA PROMOTER BINDING PROTEIN‐LIKE (SPL) family proteins are plant‐specific transcription factors, that share a highly conserved zinc ion‐containing DNA binding domain named the SBP‐box (Wang *et al*., 2009; Yamasaki *et al*., 2004). In *Arabidopsis (Xie et al.,* 2006 *) and* rice (Wang *et al*., 2009), respectively, 16 and 19 SPLs have been found. These *SPLs* are conserved across monocots and eudicots (Wang *et al*., 2009), but each individual member may function divergently in the regulation of various processes. For example, *AtSPL3*/*4*/*5* redundantly regulates developmental ageing and phase transition in *Arabidopsis* (Jung *et al*., 2016; Yamaguchi *et al*., 2009); *AtSPL9* controls the initiation of cauline leaf axillary meristems in *Arabidopsis* (Tian *et al*., 2014); *AtSPL8*/*2*/*9*/*15* redundantly acts in pollen development and male fertility in *Arabidopsis* (Unte *et al*., 2003; Xing *et al*., 2010, 2013); *OsSPL13* and *OsSPL16* regulate grain size and shape in rice. *OsSPL14*, also known as *IDEAL PLANT ARCHTECTURE1* (*IPA1*), has been found to promote panicle branching while suppressing basal branch formation in rice (Jiao *et al*., 2010; Miura *et al*., 2010), an ideal situation for grain yield increase but not necessarily for biomass production. Such tiller suppression is not due to genetic inhibition of the basal bud initiation or outgrowth, but due to a delay in the time that elapses between the formation of primordia (i.e. a prolonged plastochron) and to the associated regulatory effects of leaf development (Wang and Li, 2011).

In this study, we identified three mutants with enhanced branching from the *M. truncatula Tnt1* mutant population. Molecular analysis revealed that these mutations were caused by *Tnt1* insertions in different regions of *MtSPL8*. Overexpression of *MtSPL8* in wild‐type *M*. *truncatula* resulted in a dramatic decrease in branch formation. Further analyses revealed that *MtSPL8* controlled branching by directly inhibiting axillary bud initiation. Subsequently, alfalfa *SPL8* gene (*MsSPL8*) was cloned, and the transgenic alfalfa lines with overexpression or down‐regulation of *MsSPL8* exhibited similar phenotype to what was observed in *M. truncatula* transgenics or mutants. In particular, knockdown of *MsSPL8* significantly increased biomass yield, promoted regrowth and enhanced salt and drought tolerance. Our results demonstrate simultaneous improvement of multiple important agronomic traits by genetic manipulation of a single *SPL* gene.

## Results

### An SPL gene regulates branching and shoot architecture

**Figure 1:**
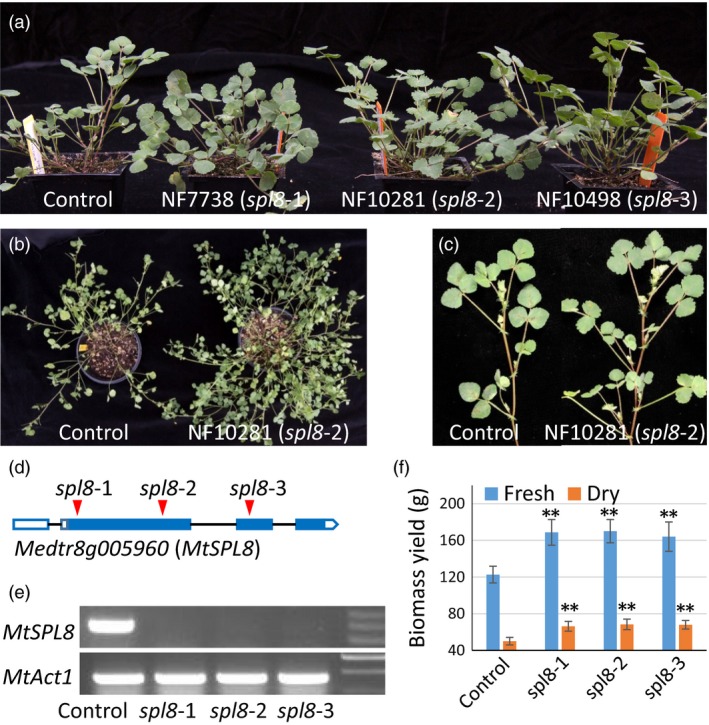
Phenotype and biomass yield of three *Tnt1* mutants of *SPL8* in *M. truncatula*. (a) Phenotype of 4‐week‐old mutant plants. (b) Eight‐week‐old plants of mutants and control. (c) Branch development in the control and spl8 mutants. (d) *Tnt1* insertion sites in *SPL8* of the three mutants. (e) Semiquantitative PCR indicates that *MtSPL8* expression is abolished in *spl8* mutants. (f) Fresh and dry biomass yields of mutant and control plants. Values represent mean ± S.D. of five biological replicates and are analyzed by Student's *t*‐test (**P <* *0.01).

To identify mutants with shoot architecture alterations, over ten thousand independent lines of *Tnt1* retrotransposon‐tagged *M. truncatula* populations were screened. Three mutant lines (NF7738, NF10281 and NF10498) were identified that exhibited enhanced branch development. The three mutants showed no obvious difference in very early development. Three weeks after sowing, these mutants started to develop more branches than control plants (ecotype R108) (Figure 1a). After 6–8 weeks, along with the initial formation of additional secondary branches the difference became more apparent as even greater numbers of secondary and tertiary branches were produced in the mutants (Figure 1b and c). Except for this phenotype, the three mutants showed no difference in other morphological traits, including seed germination, leaf pattern, leaf size, plant height, flower development, floral structure, or pod and seed shape and size.

To determine which gene(s) underlie this phenotype of enhanced branching, we first investigated the three mutants for all possible defective genes. Based on a search of the *Medicago truncatula* Mutant Database (https://medicagomutant.noble.org/mutant/database.php), we found 35, 45 and 109 potential *Tnt1* insertions in mutants NF7738, NF10281 and NF10498, respectively. By limiting the candidates to only the high confidence ones, the number of *Tnt1* insertions decreased to 14, 17 and 29 in the three mutants. Further analysis showed that only two mutated genes (*Medtr8g005960* and *Medtr1g102390*) were common to all three mutants. To verify which gene or genes caused the phenotype, each individual mutant was crossed with the wild type. The F1 plants did not show the phenotype; however, enhanced branching was observed in segregated F2 plants. Based on PCR verification (using primers across the *Tnt1* insertion sites in the two genes), we found that all the F2 plants with phenotype contained the homozygous mutation only in *Medtr8g005960*. The three homozygous mutants had *Tnt1* insertions in different exons of *Medtr8g005960* (Figure 1d). Semiquantitative PCR (using selected primers from exons 2 and 3) showed that *MtSPL8* expression was abolished in all mutants (Figure 1e). These results together suggest that only *Medtr8g005960* corresponds to the enhanced branching phenotype. Sequence BLAST analysis suggested that *Medtr8g005960* coded a *SPL* gene containing three exons (Figure 1d). In *M. truncatula* genome, a total of 11 *SPL* genes were found by BLAST analysis using the *Medicago truncatula Mt4.0v1* (https://phytozome.jgi.doe.gov/pz/portal.html#!info?alias=Org_Mtruncatula) (Figure S1). *Medtr8g005960* was named *MtSPL8* because it is closely related to *AtSPL8* in *Arabidopsis* (Figure S1). Correspondingly, the three mutants NF7738, NF10281 and NF10498 were named as *spl8‐1*,* spl8‐2* and *spl8‐3*.

The expression pattern of *MtSPL8* was analysed using the *M. truncatula* Gene Expression Atlas (https://mtgea.noble.org/v2/index.php). The expression of *MtSPL8* exhibited distinct tissue specificity. It was predominantly expressed in the vegetative bud, flower and pod. In contrast, it showed very low expression in the other organs, with almost no expression in the root (Figure S2).

### The *spl8* mutants showed significantly more lateral branching and increased biomass yield

Repeated experiments demonstrated that the *spl8* mutants began to form more branches three weeks after sowing, and the difference became more significant after 6 weeks or more (Figure 1c). To decipher details of this time‐critical factor, we investigated the development of primary, secondary and tertiary branches when the plants were 10 weeks old. At that time, both control and mutants showed nearly 50% flowering. Our data showed the three mutants had 24.5%–33.8% more primary branches than control (*P *<* *0.05, Figure S3). The difference was even greater with respect to lateral branches (secondary + tertiary). Mutants had 50.2%–58.5% more secondary branches and 131.3%–179.7% more tertiary branches (Figure S3). These results suggest enhanced branching during development.

The *spl8* mutants displayed more than 39.1% increase in fresh biomass yield (Figure 1f), and more than 32.3% increase in dry biomass (Figure 1f) due to the increased branching. The differences were highly significant (*P *<* *0.01).

### Overexpression of *SPL8* suppressed branching

**Figure 2:**
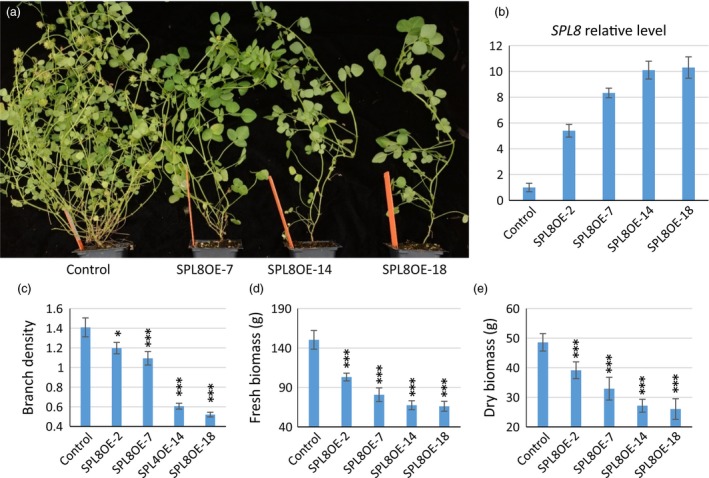
Effects of *SPL8* overexpression in *M. truncatula*. (a) Mature plants of control and *SPL8* overexpression transgenic lines (SPL8OE). (b) *SPL8* relative expression levels in transgenic plants. (c) Branch density (primary branches produced in one centimeter of main stem) of overexpression lines and control. (d) Fresh biomass yield of overexpression lines and control. (e) Dry biomass yield of overexpression lines and control. Values represent means ± S.D. of three biological replicates and are analyzed by Student's *t*‐test (*P <* *0.05, ***P <* *0.001).

To further confirm its function, *MtSPL8* was overexpressed in the wild type. Forty‐four independent antibiotic resistant plants were produced by *Agrobacterium*‐mediated transformation (Figure. S4). PCR analysis, using a forward primer designed from the CaMV35S promoter and a reverse primer designed from the 5′‐end of *SPL8*, showed bands of expected sizes in 42 transgenic events (Figure S4a). Based on *SPL8* expression level (Figure S4b), 15 lines were selected and their T1 progenies were further analysed by PCR. The results confirmed transgenic nature of the T1 plants (Figure S4c). During their entire lifespan, these transgenic plants showed dramatically decreased primary branches and lateral branches (Figure 2a) and the phenotype remained stable in the T2 plants. Notably, there were five transgenic plants that showed very few branches and especially rare lateral branches (Figure 2a. SPL8OE‐14 and 18 are two representatives of these plants). Consistently, these five plants showed the highest expression levels of *MtSPL8* (over 10‐fold more than control) among the transgenics (Figure 2b, Figure S4b). The overexpression plants were taller than the control and *spl8* mutants (Figure S5a and b); however, their branch density (branch number/1 cm main stem) and total branch numbers were dramatically decreased (Figure 2c, Figure S5c). The differences were more significant in secondary and tertiary branching than for primary branches (*P *<* *0.001 *vs*. *P *=* *0.0265). Unsurprisingly, the biomass yield in the overexpression plants was also markedly decreased (Figure 2d and e).

### *SPL8* directly regulates axillary bud initiation

**Figure 3:**
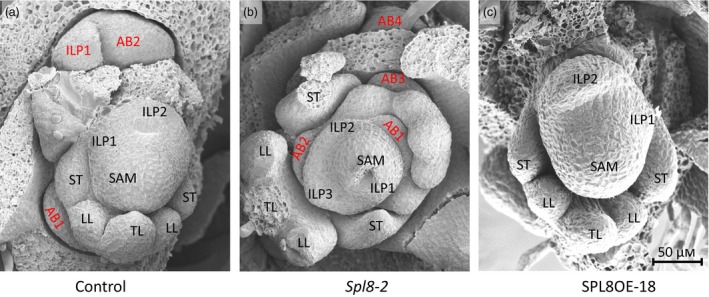
SAM development in *spl8* mutant (*spl8*‐2), control and overexpression plant (SPL8OE‐18) in *M. truncatula*. (a) SAM structure in the control. (b) SAM structure in *spl8*. (c) SAM structure in SPL8OE with complete removal of all surrounding tissue and leaflets. AB, axillary bud; ILP, incipient leaf primordia; LL, lateral leaflet primordia; TL, terminal leaflet primordia; ST, stipule primordia.

From *spl8* mutants to the overexpression plants, the results consistently indicated that *SPL8* regulates branching (Figure S5). As branches are developed from axillary buds, the formation of axillary buds was investigated using scanning electron microscope (SEM). Shoot tips of 5‐week‐old plants were harvested and examined under SEM. This observation revealed that axillary buds were formed following the elongation of leaf primordia in the control. In most cases, a well‐developed axillary bud could easily be found at the axil of the oldest leaf primordia or the youngest juvenile leaf (Figure 3a). Compared to control, bud formation in *spl8* mutants occurred much earlier, and the size of the bud appeared bigger relative to the control at the same position (Figure 3b). The results demonstrated that axillary bud initiation was significantly promoted in *spl8* mutants. In contrast, no obvious axillary buds were found in the *MtSPL8* overexpression plants (Figure 3c). To confirm this observation, we attempted to remove all juvenile leaves and other surrounding tissues, but no axillary bud was observed in the same position as that of the control (Figure 3c). Thus, overexpression of *MtSPL8* markedly inhibited axillary bud initiation.

### Global gene expression profiling of *SPL8* regulation

To understand the underlying mechanisms, shoot apical meristems (SAM) harvested from the five overexpression plants with highest *MtSPL8* expression levels (Figure S4b) and the control were subjected to microarray analysis using Affymetrix GeneChip Medicago Genome Array. Microarray analysis revealed that 199 and 176 genes were significantly down‐ and upregulated in the transgenic plants (Tables S1 and S2). MapMan analysis revealed that stress response was the first most significantly regulated biological process. Twenty‐three of the 199 down‐regulated genes were associated with abiotic and biotic stress responses, such as defensin and defensin‐like genes, production of anthocyanin pigment 1 (*PAP1*), chalcone synthase (*CHS*) and dihydroflavonol‐4‐reductase (*DFR*) genes (Table S1). Endogenous signalling was identified as the second most significant process, which included 11 gibberellin (GA) signalling genes, such as *GA2‐ox6* (encodes a major GA deactivator), *GID1L2* (encodes a GA receptor) and *GASR*s (GA responsive genes). Transcriptional regulation was the third most significant process, which included many MADSs, MYBs and WRKYs. In addition, *SCARECROW‐like 11*, the *LAS* ortholog in *Medicago*, also showed a significant difference.

To confirm the microarray results, representative genes were chosen from these biological processes for further analysis using reverse transcription quantitative PCR (RT‐qPCR) in SAM and mature leaves. The results confirmed that all of these genes showed consistent and verifiable changes in the mutants, compared to control and overexpression plants in both SAM and leaves (Figure S6).

### *SPL8* is involved in the regulation of GA signalling

Many GA signalling genes were down‐regulated by *SPL8* except for *GA2ox6* which was up‐regulated, indicating a difference in bioactive GA accumulation. SAM and mature leaves harvested from *spl8* mutants, control and overexpression plants were subjected to phytohormone quantification. The data confirmed that *spl8* mutants did accumulate higher GA_4_ than control, while the overexpression plants consistently accumulated much less. The differences were more significant in SAM than in leaf tissues (Figure S7). However, GA_1_ was undetectable in both tissues. Both GA_1_ and GA_4_ are the bioactive GAs, but each has specific predominance in different species. This result also indicates that, in *M. truncatula*, the major bioactive GA is not GA_1_ but GA_4_. Compared to GA, both IAA and ABA showed no significant difference in either SAM or leaf (Figure S7).

### Isolation of the *SPL8* gene from alfalfa

Because many genes have high sequence similarity between *M. truncatula* and alfalfa, the full‐length mRNA sequence of *SPL8* was isolated from alfalfa by 5′‐ and 3′‐RACE using primers derived from the *M. truncatula SPL8*. The resulting putative orthologous gene from alfalfa was designated as *MsSPL8*. Sequence analysis showed that there was 89.6% similarity between *MtSPL8* and *MsSPL8* (Figure S8). Analysis of deduced amino acid sequences revealed that the MsSPL8 protein contained 304 amino acids, showing 89.9% identity to MtSPL8, with identical sequences in their SBP domain (Figure S9). The MsSPL8 protein sequence was also highly similar to its putative ortholog in soybean, with only one amino acid difference in the SBP domain (Figure S9). These results suggest that SPL8 is highly conserved in legume species.

### Modification of *MsSPL8* expression significantly affects branching and biomass yield in alfalfa

To suppress its activity, a *MsSPL8*‐RNAi construct was introduced into alfalfa and 78 independent lines were produced by *Agrobacterium*‐mediated transformation. PCR analysis showed that 71 plants contained the target gene (Figure S10). RT‐qPCR analysis showed that the endogenous *MsSPL8* level decreased to various levels, with over 50% reduction in 37 of the 71 transgenics and over 90% reduction in eight of the transgenics (Figure S11). These plants showed a steady increase in branching when compared with the control or transgenic plants without significant reduction in *MsSPL8*. Two lines (MsSPL8Ri‐57 and MsSPL8Ri‐10) with more than 50% and three lines (MsSPL8Ri‐37, MsSPL8Ri‐14 and MsSPL8Ri‐53) with more than 90% reduction in *MsSPL8* level were selected for further analyses (Figure S12a and b).

**Figure 4:**
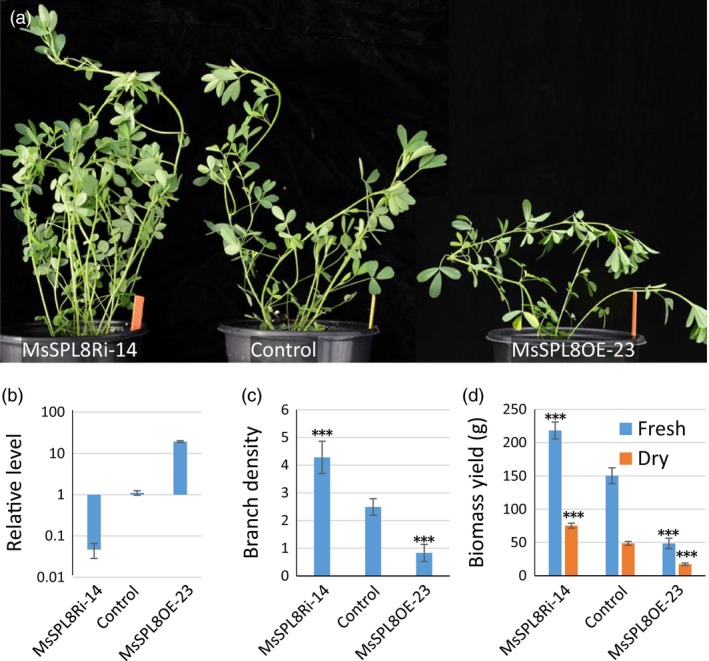
Genetic modification of *MsSPL8* significantly altered shoot architecture and biomass yield in alfalfa (*Medicago sativa*). (a) Two‐month‐old plants of control, *MsSPL8* down‐regulation (MsSPL8Ri‐14) and overexpression transgenics (MsSPL8OE‐23). (b) Relative expression levels of *MsSPL8* in the MsSPL8Ri, control and MsSPL8OE plants. (c) Branch density (the total branch numbers produced in one centimeter of main stem) of the MsSPL8Ri, control and MsSPL8OE plants. (d) Fresh and dry biomass yields (gram) of the MsSPL8Ri, control and MsSPL8OE plants. Values represent means ± S.D. of six biological replicates and are analyzed by Student's *t*‐test (***P <* *0.001).

To perform further characterization, four clonal plants were vegetatively propagated from each transgenic line using shoot cuttings. These propagated transgenic plants exhibited more branches, especially the plants with more than 90% reduction in *MsSPL8* level (Figure 4a to c, Figure S12a and b). Consistently, *MsSPL8* down‐regulation plants yielded more forage biomass than control, especially the lines with heavy down‐regulation of *MsSPL8* (Figure 4d, Figure S12c and d). The fresh and dry biomass of the heavily down‐regulated lines (MsSPL8Ri‐37, MsSPL8Ri‐14, MsSPL8Ri‐53) increased 38.5%–45.2% and 38.4%–46.5%, respectively (Figure S12c and d).

In parallel, we also overexpressed *MsSPL8* in alfalfa and produced 46 independent lines. PCR analysis showed that 44 of these were positive transgenic plants (Figure S13). These plants displayed the phenotype of decreased branches as seen in the *MtSPL8* overexpression plants (Figure S14a, Figure 2). The case was more notable in four lines with more than 10‐fold increase in *MsSPL8* level, almost no lateral branches were produced in these lines (MsSPL8OE‐23 is a representative example of the four lines, Figure S14a, Figure 4a). The extent of the decrease was negatively correlated with increased *MsSPL8* levels (Figure S14b). Consequently, forage biomass yield was dramatically decreased (Figure S14c and d).

The differences became more notable when the *MsSPL8* down‐regulation and overexpression plants were compared together (Figure 4). Side by side comparison strongly indicated that *SPL8* regulated branch development and affected biomass yield (Figure 4c and d).

### Down‐regulation of *SPL8* markedly accelerated regrowth in alfalfa

**Figure 5:**
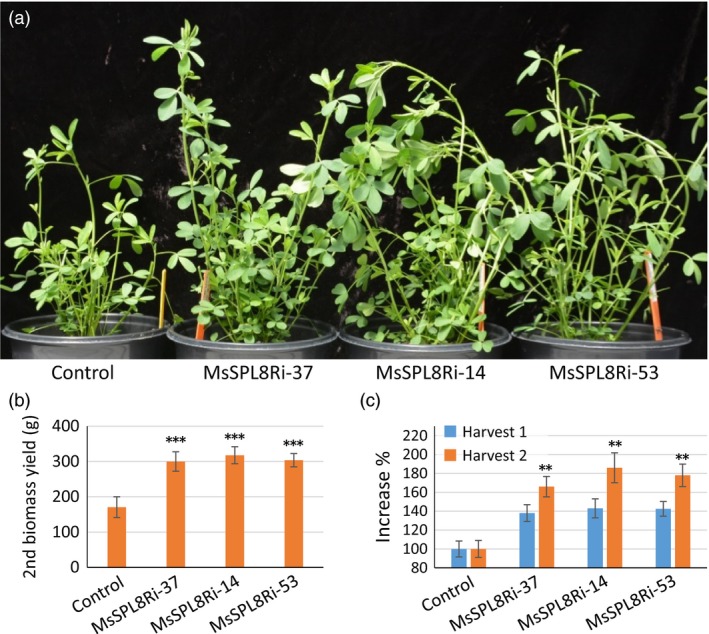
Down‐regulation of *MsSPL8* markedly accelerated regrowth in alfalfa. (a) New shoots produced after cutting from the control and *MsSPL8* down‐regulation transgenic plants (MsSPL8Ri). (b) Forage biomass (g) of the second harvest. (c) Percentage increase in biomass yield of the first and second harvests. Biomass of the second harvest is significantly higher than the first harvest in the transgenics. Values represent means ± S.D. of three biological replicates and are analyzed by Student's *t*‐test (**P <* *0.01, ***P <* *0.001).

Alfalfa plants are harvested multiple times per year. In this study, all harvests were performed when plants were at budding to early bloom stage. In three independent experiments, besides significantly improving branch formation, *MsSPL8* down‐regulation plants showed no other morphological difference, including flowering time. This allowed synchronous harvest of the control and down‐regulation plants. Interestingly, after each harvest, more shoots developed quickly in *MsSPL8* down‐regulation plants than in control plants (Figure 5a). This indicates that down‐regulation of *MsSPL8* markedly accelerated regrowth. The increase in biomass yield in *MsSPL8* down‐regulation plants was even more prominent in the second harvest (Figure 5b). With respect to the control, all three *MsSPL8* down‐regulation plants showed 37.9%–43.2% increase in biomass yield in the first harvest while the increase was 65.7%–86.3% in the second harvest. The difference between the first and second harvests was significant (Figure 5c).

The harvested biomass from three independent experiments was also analysed for forage quality. The results indicated that the *MsSPL8* down‐regulation plants had no significant difference in protein content or lignin accumulation with respect to the control. Actually, two of the three lines even showed increased total digestible nutrients and relative feed value (Table S3).

### Down‐regulation of *SPL8* expression improved abiotic stress tolerance in alfalfa

Microarray analysis of *M. truncatula* revealed that many genes were significantly regulated by *SPL8*, including *CHS*,* PAP1* and *DFR,* which are major anthocyanin genes and closely associated with stress response in plants (Cui *et al*., 2014; Gou *et al*., 2011b). To assess whether *MsSPL8* affects these genes in a similar manner in alfalfa, we analysed their expression level in 12 independent alfalfa plants. RT‐qPCR data showed that all three genes were dramatically up‐regulated in the *MsSPL8* down‐regulation plants and down‐regulated in the *MsSPL8* overexpression plants (Figure S15a).

**Figure 6:**
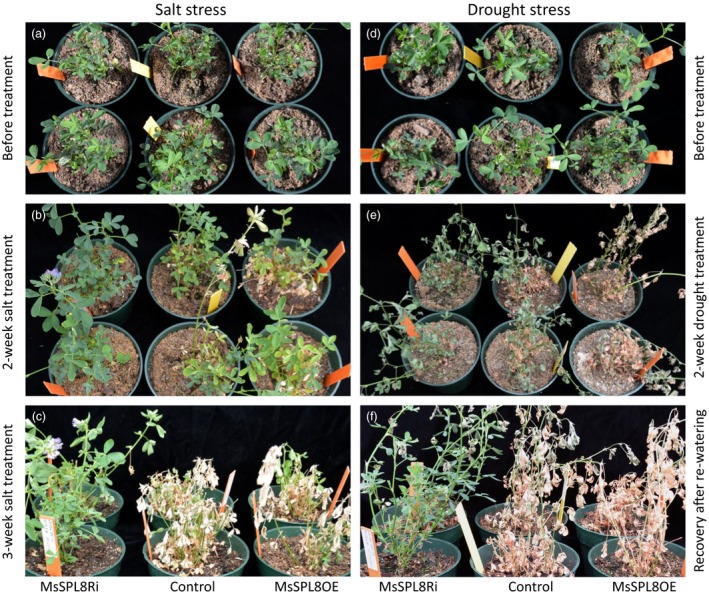
Effects of salt and drought stress on transgenic alfalfa plants down‐regulated with *MsSPL8* (MsSPL8Ri) or up‐regulated with *MsSPL8* (MsSPL8OE). (a) Plants before salt treatment. (b) Phenotype after 2‐week salt treatment. (c) Phenotype after 3‐week salt treatment. (d) Plants before drought treatment. (e) Phenotype after 2‐week drought treatment (no watering). (f) Six days of recovery after re‐watering from 2‐week drought treatment. Experiments are conducted with four biological replicates.

The effects of salt and drought stress on the transgenic plants were examined in the greenhouse. Under salt treatment (Figure 6a to c), *MsSPL8* overexpression plants began turning yellow after just 1 week. Two weeks later, the control also began turning yellow and some *MsSPL8* overexpression plants died (Figure 6b). After 3 weeks, more than 70% of overexpression plants and half of control plants were dying, by contrast, more than 90% of the *MsSPL8* down‐regulation plants still survived and some even flowered (Figure 6c, Figure S15b).

In the drought experiments (Figure 6d to f), 1 week after watering was stopped, all of the *MsSPL8* overexpression and half of the control plants wilted. Two weeks later, over half of the overexpression plants were dead and most of the control became dehydrated and dead‐like, while the *MsSPL8* down‐regulation plants just began to wilt (Figure 6e). Upon resumption of the normal watering scheme, the *MsSPL8* down‐regulation plants recovered quickly and most of them survived. In contrast, more than one‐half to two‐thirds of the control and *MsSPL8* overexpression plants were completely dead and did not recover with watering (Figure 6f, Figure S15b). These results showed that down‐regulation of *SPL8* significantly enhanced stress tolerance in alfalfa; meanwhile, overexpression of *SPL8* rendered plants more susceptible.

## Discussion

Branch development and shoot architecture are critical for plants to compete for resources and to achieve maximum light capture and carbon generation, especially because modern agricultural practice employs high‐density stands to maximize yields (McSteen, 2009; McSteen and Leyser, 2005). In forage legumes such as *M. truncatula* and alfalfa, it is known that aerial morphogenesis including branch development and shoot architecture impacts biomass yield, production persistency, lodging tolerance and forage quality (Espinoza Ldel *et al*., 2012; Julier *et al*., 2007). The main challenge is how to control branch development and optimize plant architecture. A few mapping studies showed that branch development was positively correlated with forage biomass yield, and 24 quantitative trait loci (QTLs) were mapped for branch development in four recombinant inbred line (RIL) populations of *M. truncatula* (Espinoza Ldel *et al*., 2012; Julier *et al*., 2007). Except for these few resources, limited information is available on the genetic regulation of branch development and shoot architectures in legumes.

Sixteen *SPL* genes have been identified in *Arabidopsis* (Xie *et al*., 2006). Orthologs have also been identified in various species based on their highly conserved nature across monocots and eudicots (Wang *et al*., 2009). The SPL family also shows divergent functions in the regulation of various processes, throughout phase transition, microspore development, trichome development, grain shaping and anthocyanin biosynthesis (Gou *et al*., 2011b; Jung *et al*., 2016; Si *et al*., 2016; Wang *et al*., 2015; Yu *et al*., 2010). However, very little is known about their importance to axillary bud initiation and branch formation. In rice, *OsSPL14* has been reported to decrease tiller number (basal branching) while promoting panicle branching (Jiao *et al*., 2010). However, *OsSPL14* suppresses tillering neither through axillary bud initiation nor through bud outgrowth, but by prolonging the plastochron (Wang and Li, 2011). In the current study, three mutants with enhanced branching were identified through screening a *M. truncatula Tnt1* mutant population. The enhanced branching phenotypes were caused by *Tnt1* insertions in different exons of *SPL8*. Further investigation revealed that the mutated *SPL8* enhanced branch development through the promotion of axillary bud formation. Consistently, overexpression of *SPL8* suppressed axillary bud development and subsequently inhibited branching which ultimately resulted in an alteration of shoot architecture. The *SPL8* ortholog was isolated from alfalfa. Modifying *MsSPL8* expression in alfalfa also significantly affected branching and biomass yield. The increase in biomass yield in *MsSPL8* down‐regulated alfalfa plants was 38%–43% in the first harvest, while the impact was more significant in the second harvest, with 66%–86% increase in biomass production. Alfalfa plants are typically grown in close proximity under field conditions, increased branching of the transgenics will lead to improved canopy architecture which allows better capture of sunlight. Furthermore, increased branching may allow reduced plant density and thus reduced seeding rate. It should be noted that enhanced regrowth after cutting is a particularly important trait in alfalfa. Most of the alfalfa grown in the USA is used for hay production, which requires multiple harvests per year. In some cases, alfalfa is also directly used for grazing by animals. Enhanced regrowth is very beneficial for either hay production or for grazing purpose.

Various studies indicate that *LAS*/*Ls*/*MOC1* and *LAX1*/*BA1*/*ROX* represent the two pathways that are conserved in the regulation of bud initiation in dicots and monocots (Oikawa and Kyozuka, 2009; Tanaka *et al*., 2015). The details of the underlying mechanisms are still unknown. Our microarray and RT‐qPCR analyses suggest that *SPL8* has no affect on the *Medicago LAX1* ortholog but significantly regulates the *LAS* ortholog. *AtSPL9* has been shown to directly suppress *LAS* in *Arabidopsis* (Tian *et al*., 2014). *LAS*/*Ls* orthologs encode the GRAS family nuclear proteins (including Scarecrow, SCR; Gibberellin insensitive, GAI; and repressor of gal‐3, RGA), which play a central role in the GA response and appear to be crosstalk points with other signals (Achard *et al*., 2006). A dramatic increase in GA levels was found in the *ls* mutant (Schumacher *et al*., 1999). Our data showed that GA levels were significantly higher in the *spl8* mutants while much lower in the *MtSPL8* overexpression plants, indicating that the GA signalling pathway is regulated by *SPL8* from the upstream GA receptor *GID1L2* to the downstream GA responsive genes *GRASs*. GA receptor perceives and binds endogenous GA, and the binding then induces the formation of GID1–GA–DELLA protein complex and finally triggers the subsequent signal transduction (Shimada *et al*., 2008). Furthermore, *GA2‐ox6* is significantly up‐regulated by *SPL8*. GA2‐oxidase (GA2‐ox) is the major GA deactivation enzyme (Yamaguchi, 2008). Overexpression of *GA2‐ox* resulted in a dramatic decrease of bioactive GAs (Gou *et al*., 2010, 2011a). Taken together, these evidences consistently suggest that *SPL8* regulation likely proceeds by inhibiting *LAS*/*Ls*/*MOC1* pathway. *SPL8* regulation appears to crosstalk with GA signalling, and *GA2‐ox* is possibly the key node of this crosstalk regulation. Further study of the interaction between *SPL8*,* LAS* and *GA2‐ox* may enable us to understand this mechanism in detail.

Alfalfa is the fourth most widely grown crop in the United States behind only corn, wheat and soya beans. Compared to the other crops, alfalfa has a relatively high level of drought tolerance (Zhang *et al*., 2005). Even so, drought tolerance is still a key challenge in improving alfalfa productivity (Arshad *et al*., 2017; Lei *et al*., 2017). Salinity is a major threat to alfalfa production (Arshad *et al*., 2017). Previous studies have gained some valuable information. For example, overexpression of *WXP1*, a gene related to wax accumulation, enhanced drought and dehydration tolerance in alfalfa, but the transgenic plants also showed moderately slow growth (Zhang *et al*., 2005). Overexpression of *miR156* conferred salt tolerance in alfalfa, but plant height and flowering time were negatively affected in the transgenics (Arshad *et al*., 2017). In our study, the down‐regulation of *MsSPL8* markedly enhanced drought and salt tolerance in alfalfa without any negative morphological or developmental changes. In future studies, these very promising results in controlled growth conditions will have to be tested and confirmed under field conditions.

In summary, our study revealed a new mechanism for regulating branch development and shoot architecture in the model legume *M. truncatula*, and we have successfully applied this knowledge to alfalfa improvement. This study demonstrated that down‐regulation of *MsSPL8* significantly enhanced branching by promoting axillary bud formation and, consequently, increased forage biomass yield and promoted regrowth after cutting. Furthermore, down‐regulation of *MsSPL8* also notably enhanced salt and drought tolerance in transgenic alfalfa. Results from this research offer a valuable approach to simultaneously improve biomass production and abiotic stress tolerance in plants. This study illustrates how knowledge gained from a model system can be used to genetically improve a commercial crop.

## Experimental procedures

### Plant materials and growth conditions

*Medicago truncatula* ecotype R108 was used as the wild type. Generation of the *M. truncatula Tnt1* insertional mutant population was described previously by Tadege *et al*. (2008). Mutant and wild‐type seeds were scarified with concentrated sulphuric acid and treated at 4 °C for 5 days on filter paper. Small plantlets were transferred to Metro‐Mix 830 soil mix and grown in the greenhouse at 24/22 °C (day/night) temperature with 16 h light (390 μE/m^2^/s). An alfalfa (*Medicago sativa*) genotype, Regen SY‐4D, was used for *Agrobacterium tumefaciens*‐mediated transformation to produce transgenic plants (Fu *et al*., 2015). Both transgenic and wild‐type alfalfa plants were vegetatively propagated using shoot cuttings. All plants were grown in the greenhouse at 24/22 °C (day/night) temperature with 16 h light (390 μE/m^2^/s).

### Screening of *M. truncatula* branching mutants and cloning of *SPL8*

The three mutant lines, NF7738, NF10281 and NF10498, were identified from a *M. truncatula Tnt1*‐insertion population based on enhanced branch development. *Tnt1* flanking sequences of the mutants were found by searching the *Medicago truncatula* Mutant Database (https://medicago-mutant.noble.org/mutant/database.php). The putative *Tnt1* flanking sequences were further verified by PCR amplification (using primers identified from the *M. truncatula* genome sequence that spanned across the *Tnt1* insertion sites). The PCR products were purified and cloned into pGEM‐T Easy Vector (Promega) and sequenced using Sanger dideoxy sequencing. The flanking sequences were BLAST searched against the *M. truncatula* genome sequence at the NCBI database. The genomic sequence of *SPL8* was obtained from the *M. truncatula* R108 database (http://www.medicagohapmap.org/tools/r108_blastform).

For overexpression, the coding sequences of *SPL8* were obtained through RT‐PCR amplification using primers MtSPL8‐F and MtSPL8‐R (Table S4). The fragment was inserted into pENTR/D‐TOPO cloning vector (Invitrogen) and transferred into the pEarleyGate 100 gateway vector (driven by CaMV35S promoter) by attL/attR recombination reactions (Invitrogen). The verified constructs were used to transform *M. truncatula* ecotype R108 using leaf explants (Crane *et al*., 2006). PCR analysis of the regenerated plants was carried out using a forward primer selected from the CaMV35S promoter (35Spromoter‐F) and a reverse primer selected from the 5′‐end of MtSPL8 (MtSPL8‐R1) (Table S4).

### Gene expression quantification

Reverse transcription quantitative PCR (RT‐qPCR) was performed to analyse transcript abundance of various genes. Total RNA was extracted from various tissues by TRI‐Reagent (Invitrogen) and subjected to reverse transcription with Superscript III Kit (Invitrogen). SYBR Green (Applied Biosystems, Foster City, CA) was used as the reporter dye. The primers used for RT‐qPCR are listed in Table S4. *M. truncatula* actin2 gene (TC107326) was used as an internal control. The normalized data were analysed using Student's *t*‐test.

### Microarray analysis

Total RNA samples from three biological replicates of the selected *MtSPL8* overexpression transgenic events and the wild‐type R108 were extracted from shoot apical meristems of 6‐week‐old plants using Spectrum™ Plant Total RNA Kit (Sigma‐Aldrich). 500 ng RNA was amplified and labelled using the GeneChip 3′ IVT Express Kit (Affymetrix, Santa Clara, CA) and hybridized to *M. truncatula* Affymetrix chips. Data normalization was conducted using the robust multi‐array average (RMA). Data analysis of differentially expressed probe sets on the chip was performed by associative analysis as described previously (Dozmorov and Centola, 2003). Hierarchical analysis was used to identify genes with a positive correlation between phenotype and gene expression.

### Characterization of plant growth and development

Branch numbers were measured from three biological replicates of each tested line when the plants were 10 weeks old. Fresh biomass was measured when plants were in the budding to early bloom stage (up to 10% in bloom). The harvested biomass was dried in an oven at 45 °C for 96 h before measuring the dried biomass. The data were analysed using Student's *t*‐test.

### Microscopy analysis and photography

Vegetative bud samples were harvested and immediately fixed in 3% glutaraldehyde (in 25 mm phosphate buffer (pH 7.0) overnight and dehydrated in graded ethanol series. The fixed and dried samples were observed using Hitachi TM‐3000 scanning electron microscope (SEM). Light microscopy was performed using a Nikon SMZ 1500 stereomicroscope.

### Phytohormone quantification

Meristem tissues of vegetative buds and mature leaves were harvested from wild‐type R108, *spl8* mutants and *SPL8* overexpression transgenic lines when plants were 6 weeks old. Harvested samples were frozen in liquid N_2_ and ground immediately. Fifty mg of each sample was applied for hormone quantification with HPLC‐MS analysis as described by Pan *et al*. (2010).

### Isolation of *MsSPL8* and creation of *MsSPL8* modified transgenic alfalfa

Purified mRNA from vegetative buds of the alfalfa genotype, Regen SY‐4D, was used for cDNA synthesis. The full‐length mRNA sequence of *MsSPL8* was isolated from the alfalfa cDNA by 5′‐ and 3′‐RACE using primers produced from *M. truncatula SPL8* (Table S4).

To knockdown *MsSPL8* in alfalfa, a 443‐bp fragment of *MsSPL8* was PCR‐amplified from alfalfa cDNA using primers MsSPL8Ri‐F and MsSPL8Ri‐R (Table S4). The fragment was inserted into pENTR/D‐TOPO cloning vector (Invitrogen) and transferred into the pANDA35HK vector (Li *et al*., 2010) by attL/attR recombination reactions (Invitrogen). For overexpression, the coding sequence of *MsSPL8* was obtained through RT‐PCR amplification using primers MsSPL8cDNA‐F and MsSPL8cDNA‐R (Table S4) and cloned into the pEarleyGate 100 gateway vector driven by CaMV35S promoter. The verified constructs were transferred into *Agrobacterium* strain EHA105 using the freezing/heat‐shock method. Transgenic alfalfa plants were obtained by *Agrobacterium*‐mediated transformation as previously reported (Fu *et al*., 2015). PCR analysis of the regenerated plants was carried out using a forward primer selected from the CaMV35S promoter (35Spromoter‐F) and a reverse primer selected from the 5′‐end of MsSPL8 (MsSPL8‐R1).

### Forage analysis of transgenic alfalfa lines

Transgenic and control alfalfa plants were grown in the soil with full nutrition. Two‐month‐old plants were harvested, and fresh biomass yield was measured immediately. The materials were dried in an oven at 45 °C for 96 h to measure dried biomass. The samples were then ground through a Thomas‐Wiley Laboratory Mill (Lehman Scientific) with a 1‐mm sieve. Near‐infrared reflectance spectroscopy (NIRS) was performed using a Foss NIRS 6500 monochromator with a scanning range of 1100–2500 nm (Foss NIR Systems). Each sample was scanned eight times, and the average spectra were used for calibration. Mathematical and statistical treatments of all spectra were performed with WinISI III calibration development software (Foss NIR Systems). The existing commercial NIRS prediction equations (07AHY50) developed by the NIRS Forage and Feed Testing Consortium were employed to calculate quality characteristics of alfalfa. The precision of NIRS has been assessed by regression analysis of the predicted values and actual determined values. All data were analysed using the SAS GLM procedure (SAS Institute). Statistical significance was determined by Student's *t*‐test.

### Drought and salt treatments

Both transgenic and control alfalfa plants were propagated using shoot cuttings. Uniformed seedlings were transplanted to 4.5‐inch pots filled with Metro‐Mix 830 soil mix and grown in the greenhouse. Three weeks later, well‐established and similarly sized plants (with eight replicates for each experiment) were selected from each line for two kinds of treatments. For salt treatment, all pots were completely soaked in 1.5% NaCl solution for 3 h every 4 days for 3 weeks. For drought treatment, all plants were completely soaked in water for three hours, and then watering was withheld for 2 weeks before re‐watering. The experiments were repeated at least three times for all measurements.

## Supporting information


**Figure S1** Phylogenetic analysis of SPL orthologs in *Arabidopsis,* rice and *M. truncatula*.
**Figure S2** Expression patterns of *SPL8* in *M. truncatula*, produced from the *M. truncatula* Gene Expression Atlas (https://mtgea.noble.org/v2/index.php).
**Figure S3** Comparison of branch development between control and the three *Tnt1* mutants of *SPL8* in *M. truncatula*.
**Figure S4** PCR analysis of regenerated *M. truncatula* plants after *Agrobacterium*‐mediated transformation with the *SPL8* overexpression vector.
**Figure S5** Branching and shoot architecture were significantly altered in *SPL8* knockout mutants and overexpression transgenics in *M. truncatula*.
**Figure S6** Relative expression levels of candidate genes in *spl8* mutants, control and *SPL8* overexpression lines (SPL8OE) in *M. truncatula*.
**Figure S7** Phytohormone accumulation in *spl8* mutants, control and *SPL8* overexpression lines (SPL8OE).
**Figure S8** Sequence comparison of *MsSPL8* (*M. sativa* SY4D) with *SPL8* (*M. truncatula* R108).
**Figure S9** Amino acid alignment of MsSPL8 and SPL8 proteins and their ortholog in soybean (*Glycine max*).
**Figure S10** PCR analysis of regenerated alfalfa plants after *Agrobacterium*‐mediated transformation with the *MsSPL8* RNAi vector.
**Figure S11** Relative expression levels of *MsSPL8* in the transgenic downregulation plants of alfalfa (MsSPL8Ri).
**Figure S12** Transgenic alfalfa showing increased biomass phenotype with downregulation of *MsSPL8*.
**Figure S13** PCR analysis of regenerated alfalfa plants after *Agrobacterium*‐mediated transformation with the *MsSPL8* overexpression vector.
**Figure S14** Transgenic alfalfa showing decreased biomass phenotype with overexpression of *MsSPL8*.
**Figure S15** Effects of abiotic stresses on *MsSPL8* transgenic alfalfa plants.

**Table S1** Downregulated genes with abundance changed more than 2.5 folds in SPL8 overexpression plants relative to the control.
**Table S2** Upregulated genes with abundance changed more than 2.5 folds in SPL8 overexpression plants relative to the control.
**Table S3** Forage quality analysis of alfalfa plants.
**Table S4** Primers used in this study.
