# Supplementary material for: From model to crop: functional characterization of SPL8 in M. truncatula led to genetic improvement of biomass yield and abiotic stress tolerance in alfalfa
Source: Plant Biotechnol J. 2017 Oct 17;16(4):951–62. doi: 10.1111/pbi.12841 (PMC5866946; doi:10.1111/pbi.12841)
Supplement: Supplementary file 1 — Figure S1 Phylogenetic analysis of SPL orthologs in Arabidopsis, rice and M. truncatula. Figure S2 Expression patterns of SPL8 in M. truncatula, produced from the M. truncatula Gene Expression Atlas (https://mtgea.noble.org/v2/index.php). Figure S3 Comparison of branch development between control and the three Tnt1 mutants of SPL8 in M. truncatula. Figure S4 PCR analysis of regenerated M. truncatula plants after Agrobacterium‐mediated transformation with the SPL8 overexpression vector. Figure S5 Branching and shoot architecture were significantly altered in SPL8 knockout mutants and overexpression transgenics in M. truncatula. Figure S6 Relative expression levels of candidate genes in spl8 mutants, control and SPL8 overexpression lines (SPL8OE) in M. truncatula. Figure S7 Phytohormone accumulation in spl8 mutants, control and SPL8 overexpression lines (SPL8OE). Figure S8 Sequence comparison of MsSPL8 (M. sativa SY4D) with SPL8 (M. truncatula R108). Figure S9 Amino acid alignment of MsSPL8 and SPL8 proteins and their ortholog in soybean (Glycine max). Figure S10 PCR analysis of regenerated alfalfa plants after Agrobacterium‐mediated transformation with the MsSPL8 RNAi vector. Figure S11 Relative expression levels of MsSPL8 in the transgenic downregulation plants of alfalfa (MsSPL8Ri). Figure S12 Transgenic alfalfa showing increased biomass phenotype with downregulation of MsSPL8. Figure S13 PCR analysis of regenerated alfalfa plants after Agrobacterium‐mediated transformation with the MsSPL8 overexpression vector. Figure S14 Transgenic alfalfa showing decreased biomass phenotype with overexpression of MsSPL8. Figure S15 Effects of abiotic stresses on MsSPL8 transgenic alfalfa plants. [file PBI-16-951-s002.pdf]

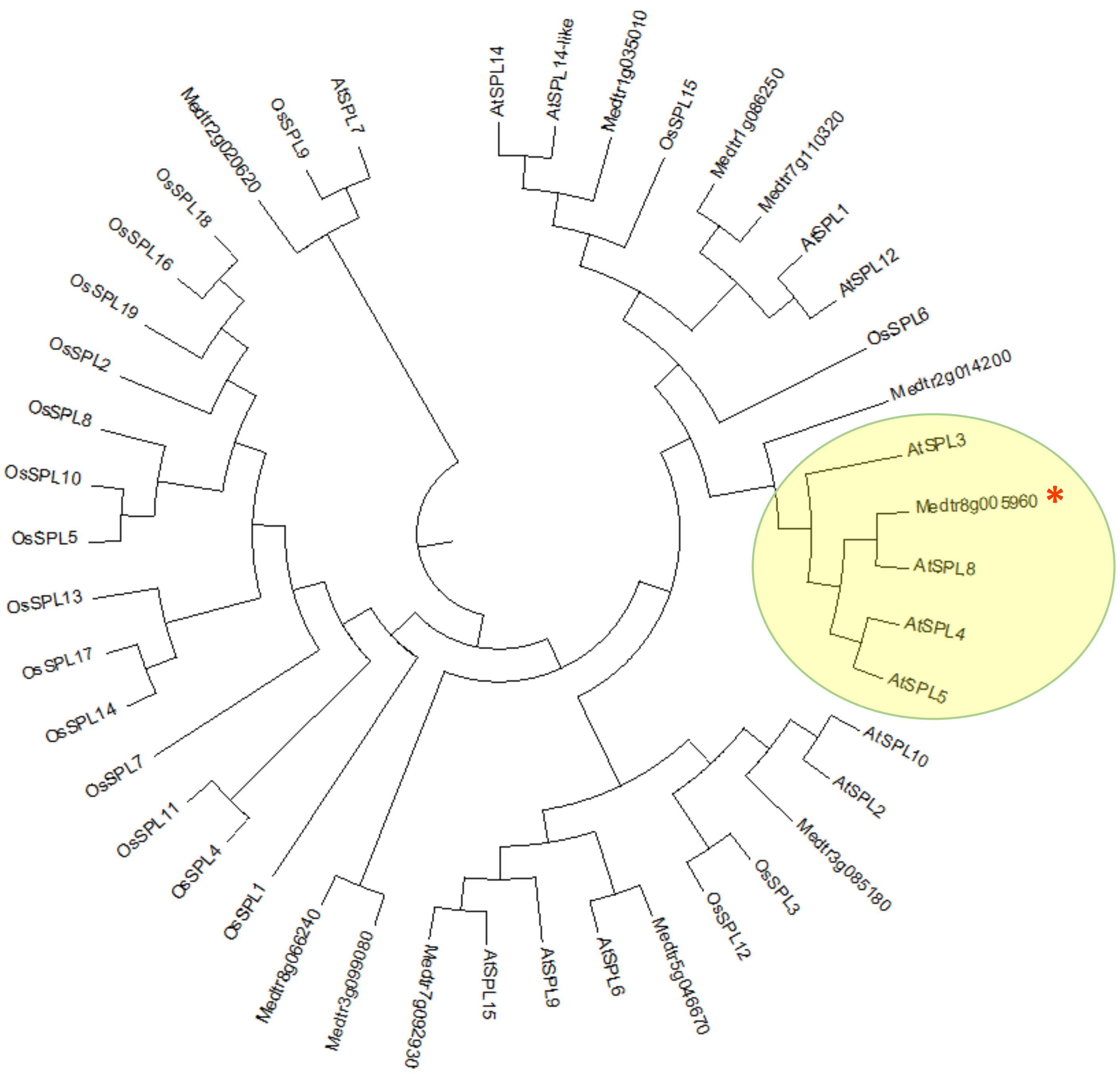

Figure S1. Phylogenetic analysis of SPL orthologs in *Arabidopsis,* rice and *M. truncatula*. The analysis indicated that *M. truncatula* SPL8 (*Medtr8g005960,* red star) is a homolog of AtSPL8 and is also close to AtSPL3, 4 and 5.

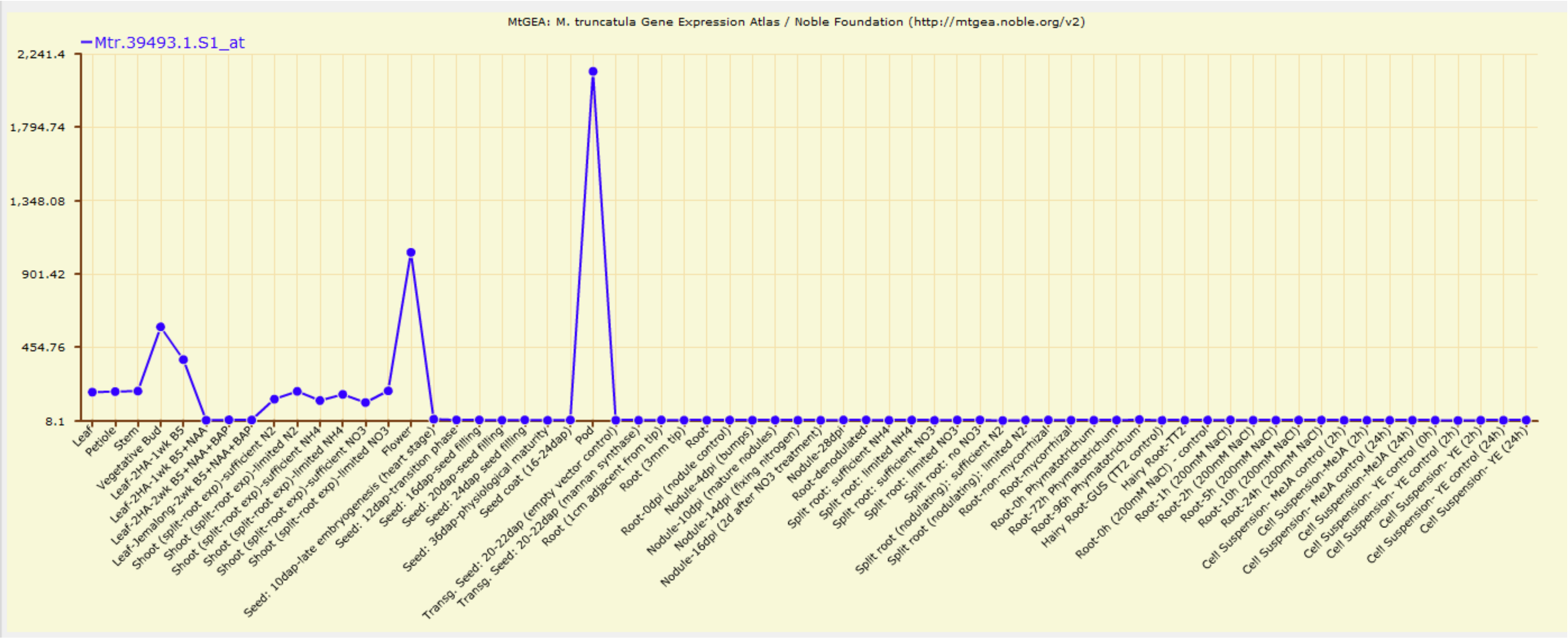

Figure S2. Expression patterns of *SPL8* in *M. truncatula*, produced from the *M. truncatula* Gene Expression Atlas (https://mtgea.noble.org/v2/index.php).

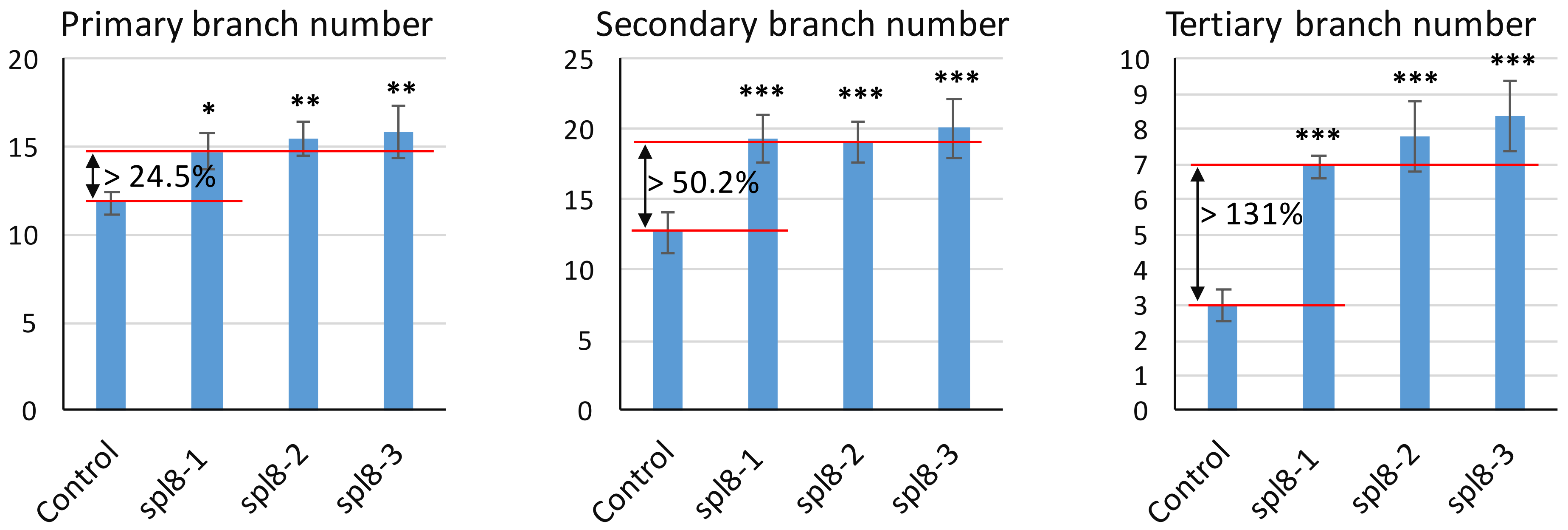

Figure S3. Comparison of branch development between control and the three *Tnt1* mutants of *SPL8* in *M. truncatula*. Values represent means ± S.D. of three biological replicates and are analyzed by Student's t-test (* $P < 0.05$, ** $P < 0.01$, *** $P < 0.001$).

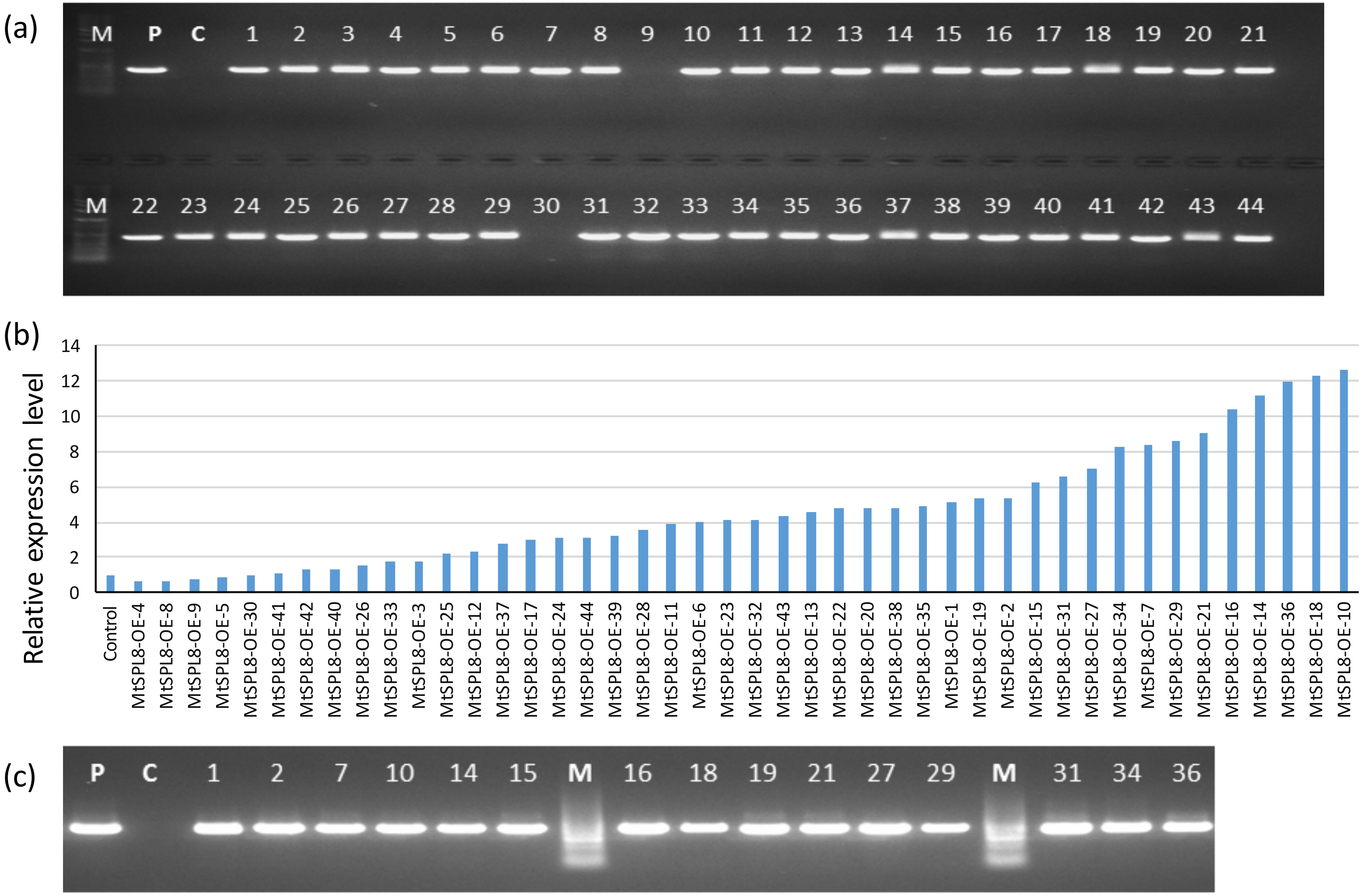

Figure S4. PCR analysis of regenerated *M. truncatula* plants after *Agrobacterium*-mediated transformation with the *SPL8* overexpression vector. (a) PCR screening of regenerated T0 plants using promoter and target gene-specific primers. (b) Relative expression levels of *SPL8* in the transgenic plants. Expression is shown relative to that of the control, which is set to 1. (c) PCR verification of 15 T1 transgenic plants. C, wild-type control; M, PCR 50-2,000bp marker (Sigma); P, plasmid DNA as positive control. Numbers represent individual regenerated plants.

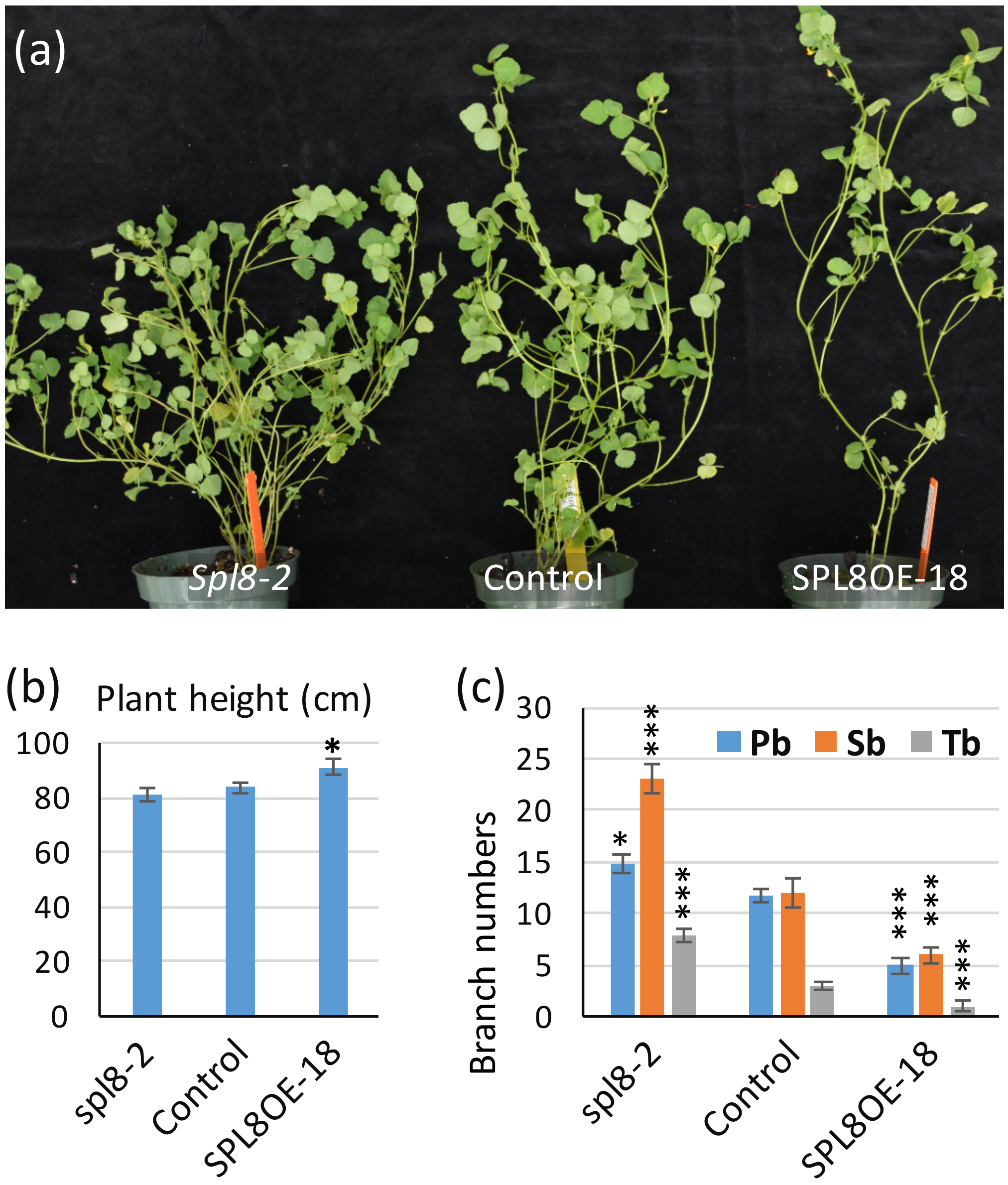

Figure S5. Branching and shoot architecture were significantly altered in *SPL8* knockout mutants and overexpression transgenics in *M. truncatula*. (a) Two-month-old plants of *SPL8* knockout mutant (*spl8-2*), control and overexpression transgenics (SPL8OE-18). (b) Plant height of *spl8-2*, control and SPL8OE-18. (c) Total number of various branches that are produced in each main stem. Pb, primary branch. Sb, secondary branch. Tb, tertiary branch. Values represent means ± S.D. of three biological replicates and are analyzed by Student's t-test (* $P < 0.05$, *** $P < 0.001$).

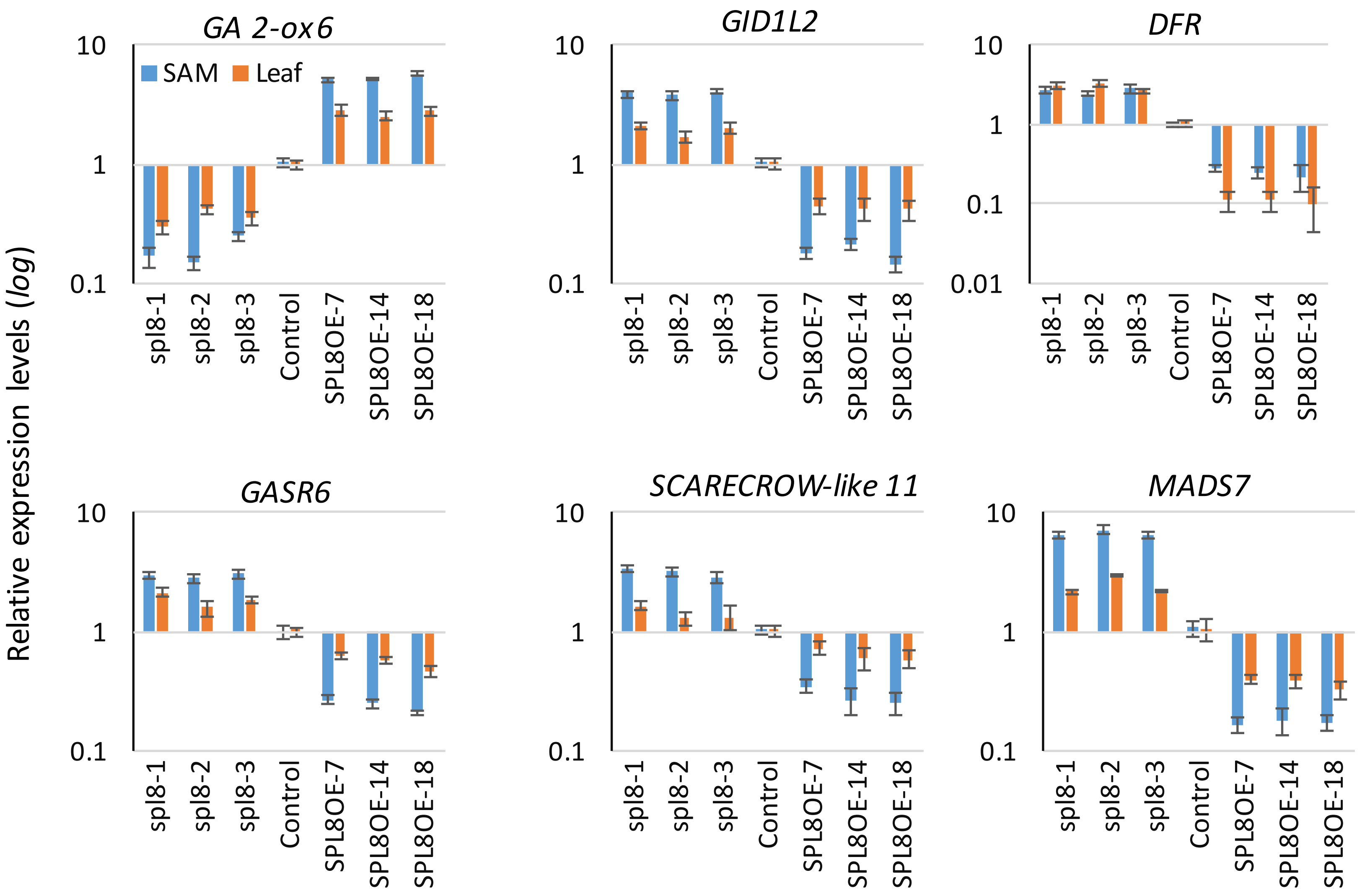

Figure S6. Relative expression levels of candidate genes in *spl8* mutants, control and *SPL8* overexpression lines (SPL8OE) in *M. truncatula*. Values represent means ± S.D. of three biological replicates.

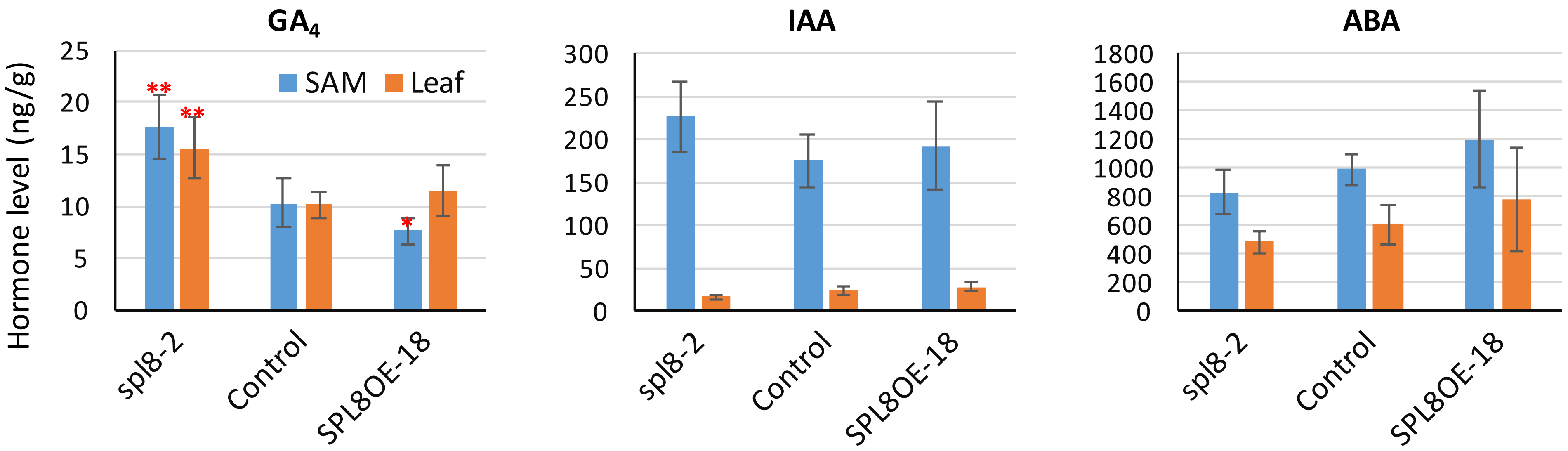

Figure S7. Phytohormone accumulation in *spl8* mutants, control and *SPL8* overexpression lines (SPL8OE). Values represent means ± S.D. of five biological replicates and are analyzed by Student's t-test (* $P < 0.05$, ** $P < 0.01$).

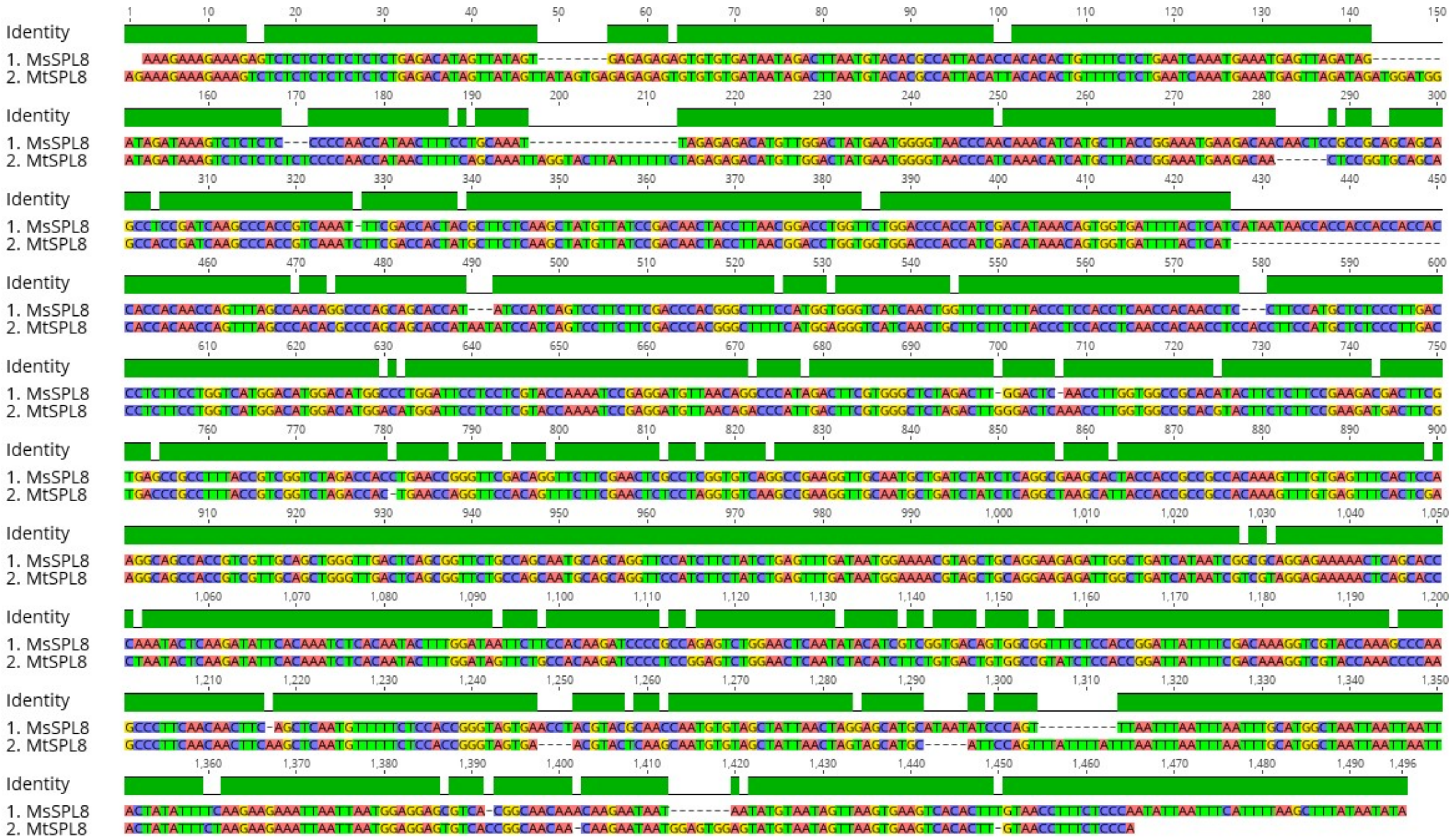

Figure S8. Sequence comparison of *MsSPL8* (*M. sativa* SY4D) with *SPL8* (*M. truncatula* R108).

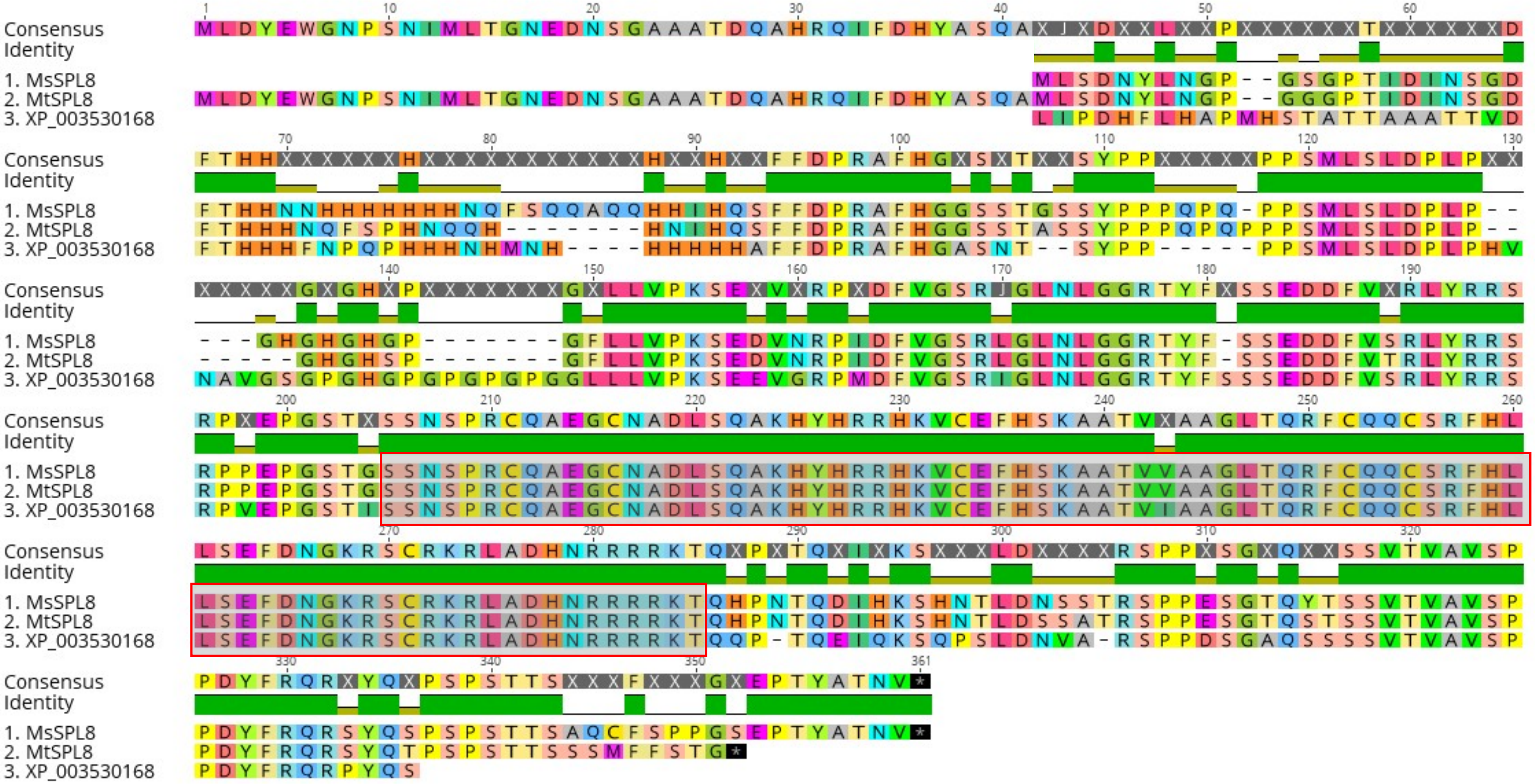

Figure S9. Amino acid alignment of MsSPL8 and SPL8 proteins and their ortholog in soybean (*Glycine max*). The red box highlights the SBP domain (206-285). *M. truncatula* SPL8 and MsSPL8 have 89.9% similarity.

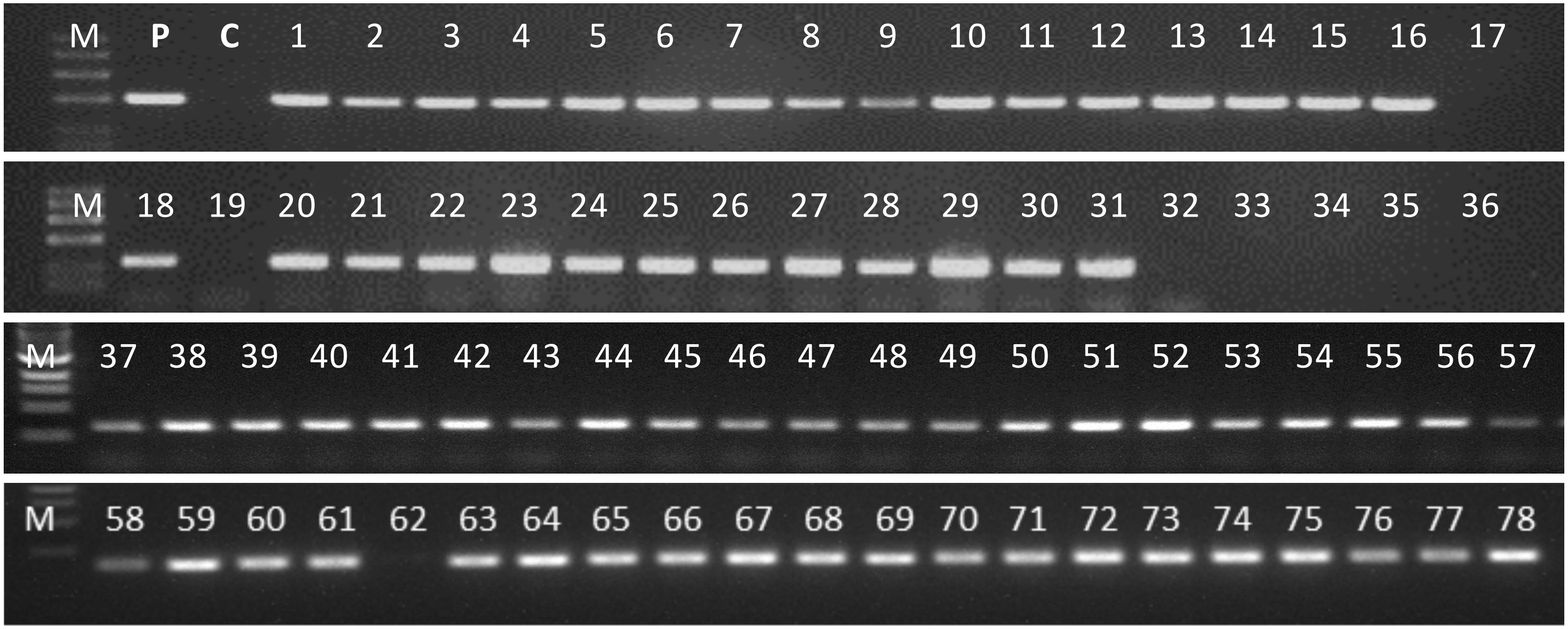

Figure S10. PCR analysis of regenerated alfalfa plants after *Agrobacterium*-mediated transformation with the *MsSPL8* RNAi vector. A pair of promoter and target gene-specific primers were used for PCR analysis. C, wild-type control; M, PCR 50-2,000bp marker (Sigma); P, plasmid DNA as positive control. Numbers represent individual regenerated plants.

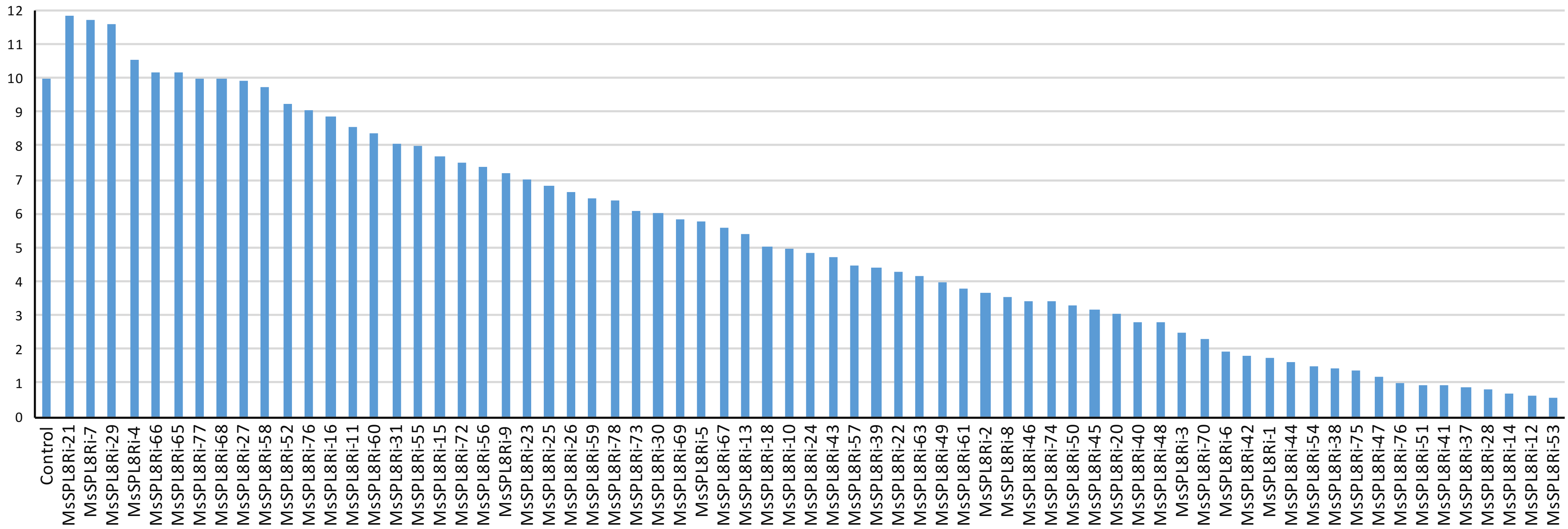

Figure S11. Relative expression levels of *MsSPL8* in the transgenic downregulation plants of alfalfa (MsSPL8Ri). Expression is shown relative to that of the control, which is set to 1.

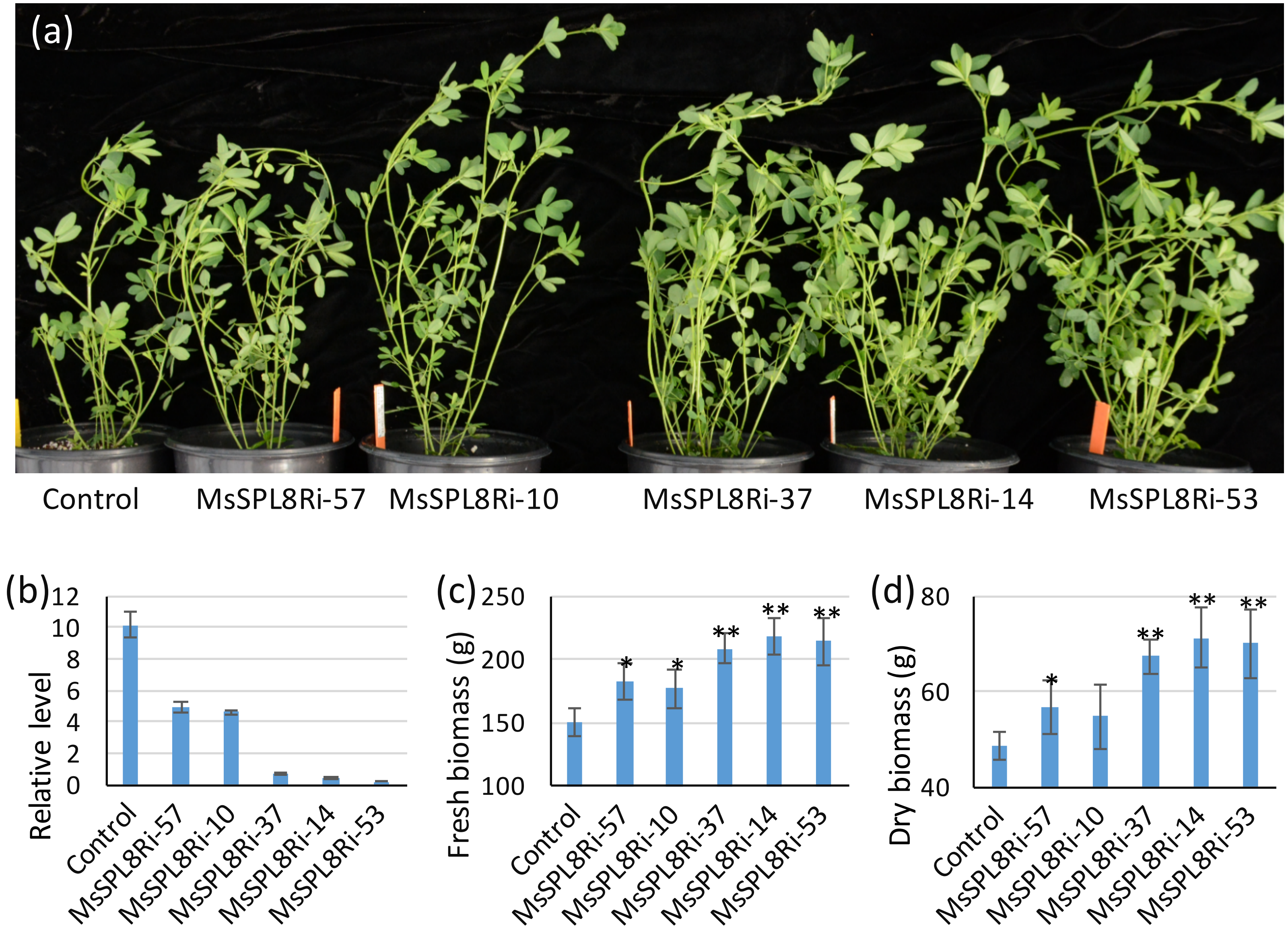

Figure S12. Transgenic alfalfa showing increased biomass phenotype with downregulation of *MsSPL8*. (a) Mature plants of control and *MsSPL8* downregulation transgenics (MsSPL8Ri). (b) *MsSPL8* relative expression levels. (c) Fresh biomass yield of control and MsSPL8Ri plants. (d) Dry biomass yield of control and MsSPL8Ri plants. Values represent means ± S.D. of three biological replicates and are analyzed by Student's t-test (* $P < 0.05$, ** $P < 0.01$).

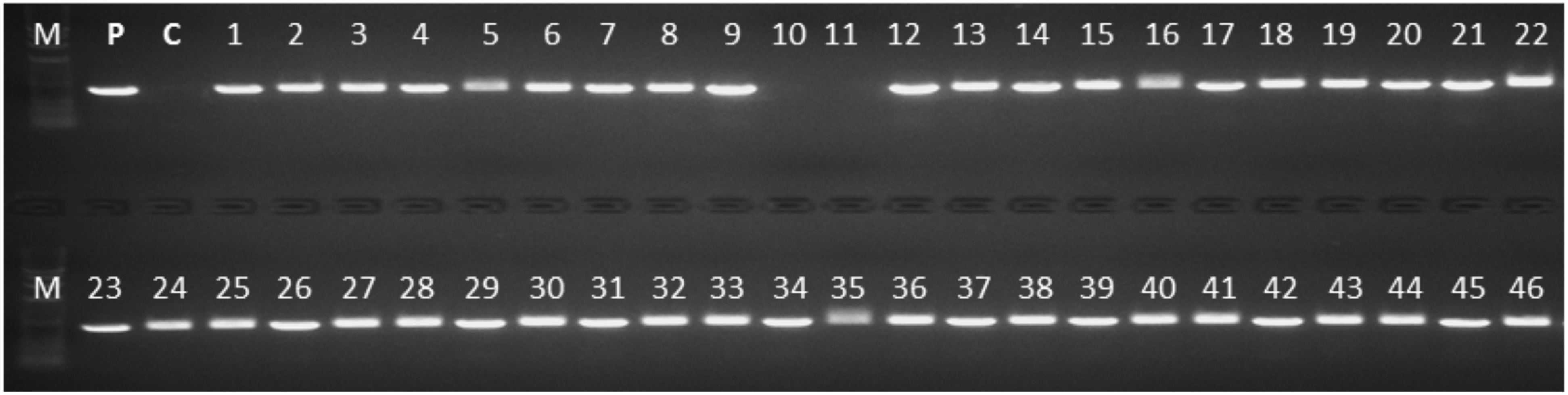

Figure S13. PCR analysis of regenerated alfalfa plants after *Agrobacterium*-mediated transformation with the *MsSPL8* overexpression vector. A pair of promoter and target gene-specific primers were used for PCR analysis, see Experimental procedures. C, wild-type control; M, PCR 50-2,000 bp marker (Sigma); P, plasmid DNA as positive control: Numbers represent individually regenerated plants.

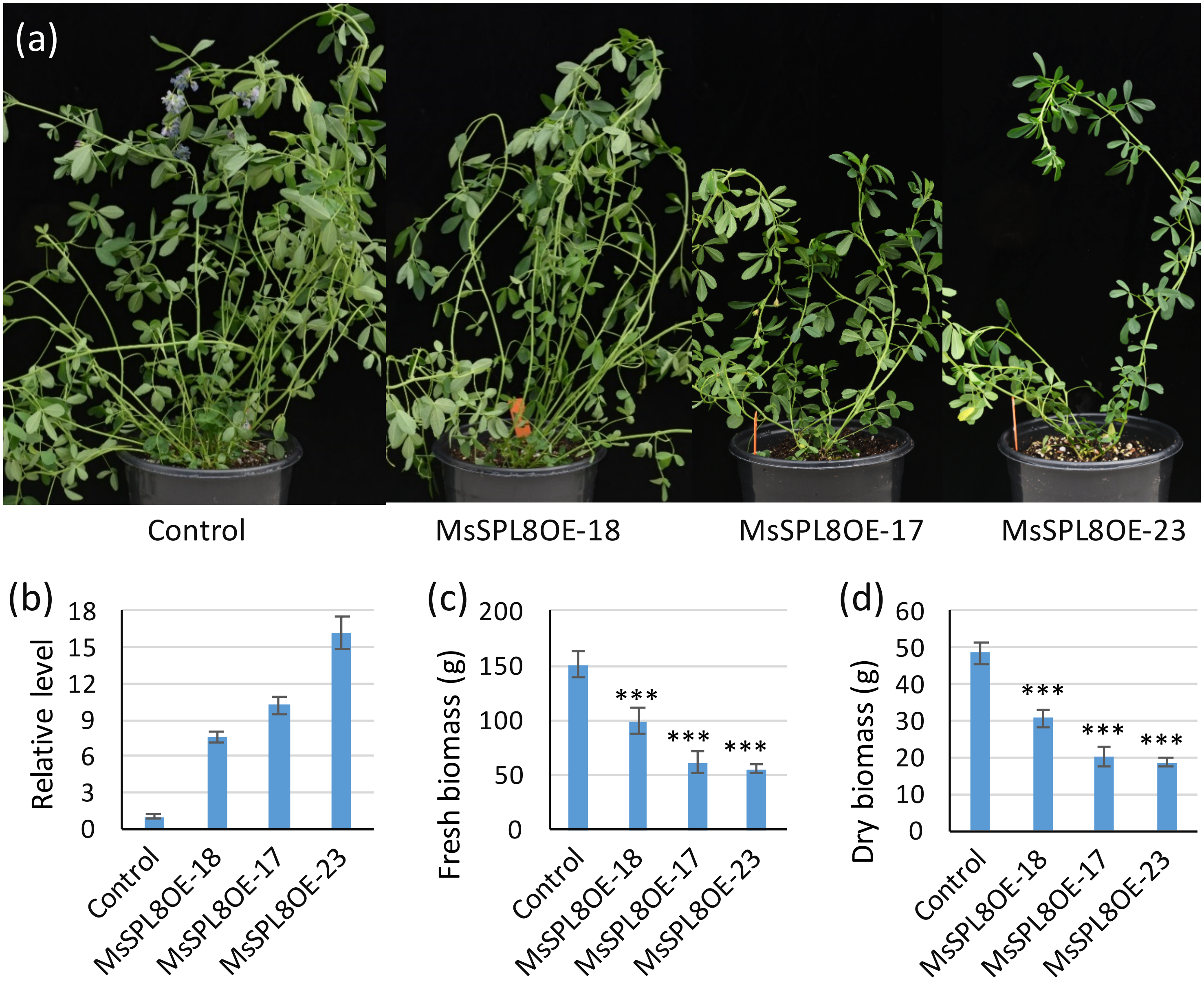

Figure S14. Transgenic alfalfa showing decreased biomass phenotype with overexpression of *MsSPL8*. (a) Mature plants of control and *MsSPL8* overexpression transgenics (SPL8OE). (b) *MsSPL8* relative expression levels. (c) Fresh biomass yield of control and MsSPL8OE plants. (d) Dry biomass yield of control and MsSPL8OE plants. Values represent means ± S.D. of three biological replicates and are analyzed by Student's t-test (*** $P < 0.001$).

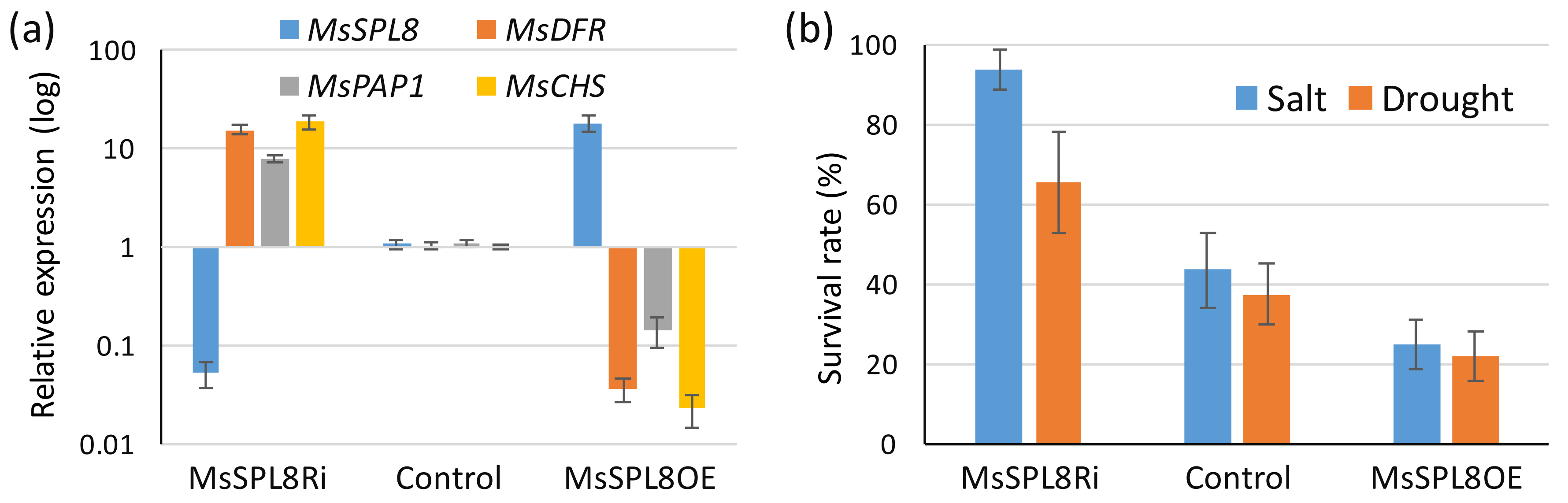

Figure S15. Effects of abiotic stresses on *MsSPL8* transgenic alfalfa plants. (a) Relative expression levels of *MsSPL8, MsCHS, MsPAP1* and *MsDFR* in different alfalfa lines. (b) Survival rate after salt and drought treatments. Values represent mean ± S.D. of four biological replicates.
